# Recent Advances in Metal–Organic Frameworks for Applications in Magnetic Resonance Imaging

**DOI:** 10.1021/acsami.2c10272

**Published:** 2022-10-14

**Authors:** Hana Bunzen, Daniel Jirák

**Affiliations:** † Chair of Solid State and Materials Chemistry, Institute of Physics, University of Augsburg, Universitätsstraße 1, D-86159 Augsburg, Germany; ‡ Department of Diagnostic and Interventional Radiology, Institute for Clinical and Experimental Medicine, Vídeňská1958/9, 140 21 Prague 4, Czech Republic

**Keywords:** metal−organic frameworks, magnetic resonance imaging, nanomedicine, theranostics, multimodal imaging

## Abstract

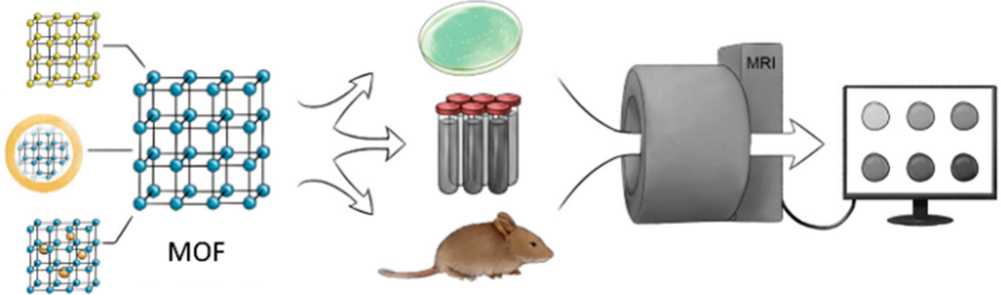

Diagnostics is an important part of medical practice. The information required for diagnosis is typically collected by performing diagnostic tests, some of which include imaging. Magnetic resonance imaging (MRI) is one of the most widely used and effective imaging techniques. To improve the sensitivity and specificity of MRI, contrast agents are used. In this review, the usage of metal–organic frameworks (MOFs) and composite materials based on them as contrast agents for MRI is discussed. MOFs are crystalline porous coordination polymers. Due to their huge design variety and high density of metal ions, they have been studied as a highly promising class of materials for developing MRI contrast agents. This review highlights the most important studies and focuses on the progress of the field over the last five years. The materials are classified based on their design and structural properties into three groups: MRI-active MOFs, composite materials based on MOFs, and MRI-active compounds loaded in MOFs. Moreover, an overview of MOF-based materials for heteronuclear MRI including ^129^Xe and ^19^F MRI is given.

## 1 Introduction

An accurate diagnosis plays a crucial role in determining a proper course of treatment. It requires collecting information from different sources, including diagnostic tests based on imaging. From this point of view, magnetic resonance imaging (MRI) is one of the most versatile, noninvasive, and nonionizing imaging techniques used in routine clinical examinations providing both anatomical and biochemical information.^1^ MRI is particularly sensitive in assessing anatomical structures, organs, and soft tissues with a high resolution for the detection and diagnosis of a broad range of pathological conditions. MR images can provide contrast between benign and pathological tissues and may be used to stage cancers as well as to evaluate the response to treatment.^2^ Although MRI is a very powerful imaging modality, in many cases, the use of contrast probes is mandatory to fully exploit the diagnostic potential of MRI by increasing the specificity and sensitivity of the method.^3^ To improve the image contrast, different compounds and materials have been examined as contrast agents and from these, few have been approved for a clinical usage.^4−7^ However, despite their success, there is still room for improvement (in order to maximize the information, which can be retracted) and thus, new compounds and materials are being constantly developed and studied as potential contrast agents.

One of the newer material groups, which have been suggested for applications in MRI, are metal–organic frameworks (MOFs). MOFs are porous crystalline coordination polymers.^8−10^ They consist of metal ions (or clusters) and bridging ligands. Due to their design variety, tunable properties, and extremely high surface area, they have been investigated in many different applications including gas storage and separation,^11−14^ catalysis,^15,16^ sensing,^17,18^ and also medical areas.^19−24^ In medicine, due their porosity (and thus a high loading capacity), they have been suggested as highly promising materials for drug delivery applications.^19−22^ Recently, also their applications in diagnostics have been examined for various imaging modalities, including fluorescence and photoacoustic imaging,^25,26^ but mainly MRI.^27,28^ The early stages of MOFs for applications in MRI were reviewed in the beginning of 2018 by Wuttke et al., who reviewed about 25 publications.^27^ Since then, the field has expanded rapidly resulting in about 161 publications (August 2022, Figure 1). In this review, we highlight the key publications from the past and focus on the latest progress of the field over the last 5 years. Moreover, only publications, which comprise MRI measurements, are included (ca. 55 publications); i.e., reports, which suggest the concept of using MOFs in MRI, but do not show any experimental data, are omitted.

**Figure 1:**
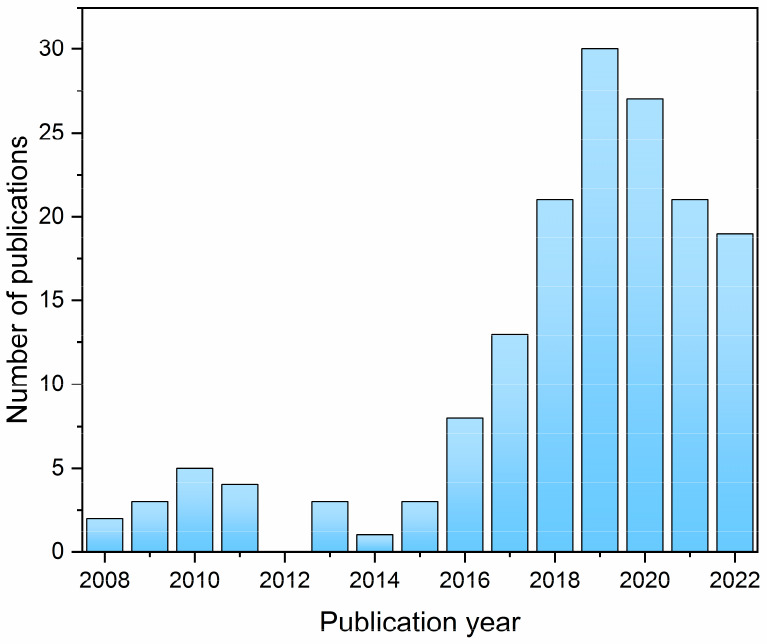
A number of publications per year containing both terms “metal-organic framework” and “magnetic resonance imaging” according to the SciFinder database (August 2022).

## 2 Basics of MRI

### 2.1 MR Phenomena

Magnetic resonance (MR) is a method based on the distribution and behavior of magnetic moments of particular isotopes in a magnetic field.^29^ As the method is nonionizing and provides information about biochemical processes in living tissue, it has become one of the most important noninvasive imaging techniques. Simply put, the principle of the MR method is based on the absorption of energy by nuclei placed in a strong static magnetic field. In general, all isotopes with a nonzero magnetic moment such as hydrogen, fluorine and phosphorus can be employed. However, for routine clinical applications, only ^1^H nuclei are used because their MR sensitivity is greater than all other nuclei. The distribution of water molecules reflects the structural composition of tissue. As changes in the water properties of tissue also closely reflect pathologic processes, this relationship is an important factor in the high success rate of MR imaging in medical applications.

The frequency of precession of isotopes depends on the magnetic field intensity of external field and on the type of nucleus, which is expressed by gyromagnetic constant γ. The external magnetic field represented by short radiofrequency pulses, the frequency of which corresponds to the Larmor frequency (in the range of 10 MHz–1 GHz), applied perpendicular to the static field, affects the magnetization vector generated by the inserted nuclei. Thus, the system of nuclei starts to absorb the energy of electromagnetic field of the radiofrequency pulses. This phenomenon is called the nuclear magnetic resonance. Depending on the radiofrequency pulse intensity and the duration, resulting magnetic moment can be flipped in any orientation, most often onto a plane that is perpendicular to external static magnetic field. Immediately after the radiofrequency pulse is applied, all excited nuclei are at the same phase and start to return to equilibrium. The return is called the relaxation process, during which the nuclei release absorbed energy in the form of electromagnetic radiation detected in the receiver coils. The induced alternating electromotive force is called the MR signal or the free induction decay signal and contains a signal from each excited nucleus and its amplitude is proportional to the number of nuclei that contribute to its formation.

### 2.2 MR Relaxation

The speed of relaxation in biological tissues is generally in the range of several milliseconds to a few seconds. As nuclei are parts of molecules, their relaxation processes depend on various factors such as temperature, magnetic field strength, chemical bonds, molecular motions, size of molecule, etc. Relaxation plays a key role in MR imaging because it affects the contrast between tissues; the difference in relaxation times makes it possible to achieve contrast in MR images. There are two independent relaxation processes, each with exponential dependence: longitudinal (T_1_) relaxation and spin–spin (T_2_) relaxation. The T_1_ relaxation can be described as an energy flow between excited spins and their external environment; the predominant mechanism of that is dipole–dipole interactions. The spin–spin relaxation occurs due to an exchange of energy between protons within the excited system; there is no energy change in the system of excited atoms. The T_2_ relaxation reflects the speed of loss of the measurable macroscopic magnetization in the plane perpendicular to the external static magnetic field. It also indicates the magnetic inhomogeneities inside the excited sample because they can affect the speed of phase coherence lose significantly. In practice, we must take into account the local magnetic field nonuniformities resulting from intrinsic defects in the magnet itself or from susceptibility-induced field distortions produced by the tissue or other materials placed within the field. Therefore, an effective T_2_* (also called T_2_ star) relaxation time (always shorter than T_2_), which covers all sources of field inhomogeneities across a voxel, is often used.

### 2.3 MR Imaging

**Figure 2:**
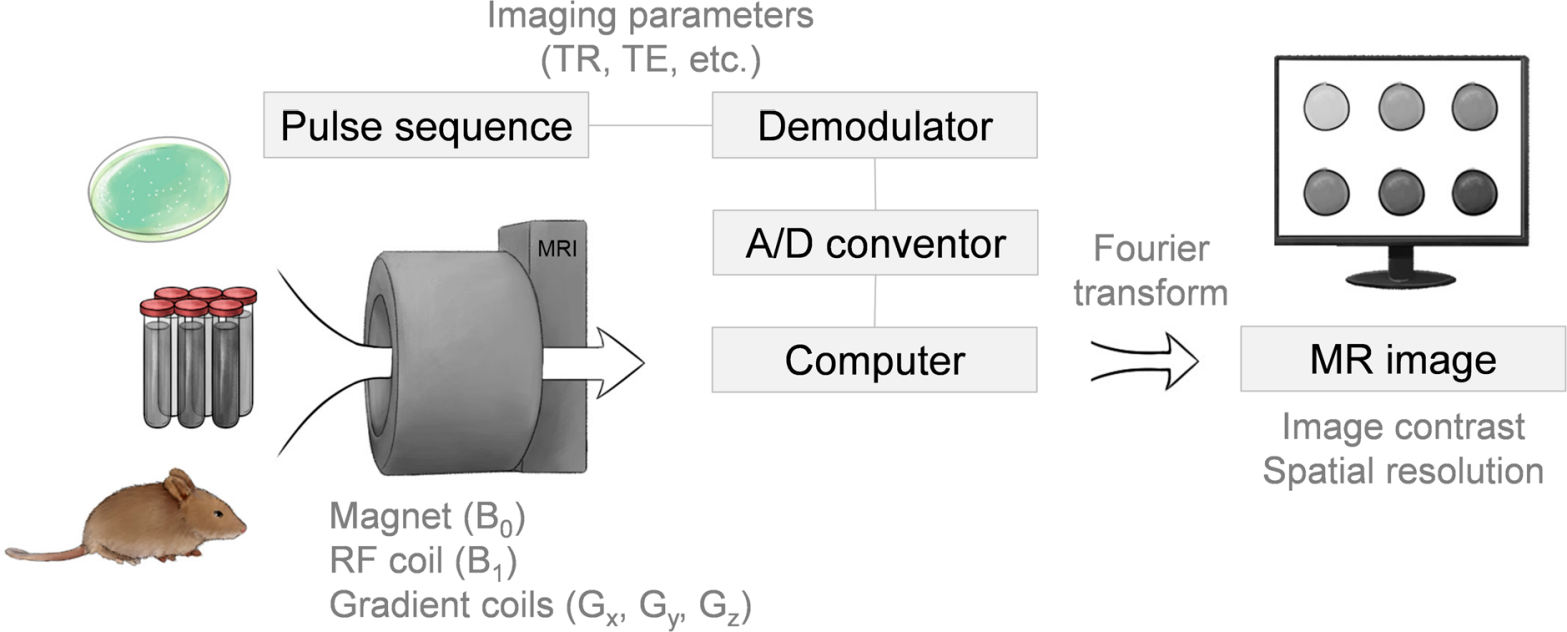
Scheme of a typical MRI systems. A specimen is placed in a magnetic field (B_0_), then a radio frequency (RF) coil produces a B_1_ field that changes the direction of the magnetization in a manner prescribed by the pulse sequence. Spatial localization is generated with the use of the gradient coils. Variation of the imaging parameters such as the repetition time (TR) and echo time (TE) in the pulse sequence provides the basis for different contrast mechanisms.

MR images are characterized by signal intensity and contrast, which are affected by the T_1_ and T_2_ relaxation as well as proton density. This means that for the same object from the same area, different MR contrasts are produced according to the dominant influence (weighting), i.e., proton density or T_1_ and T_2_ (T_2_*) relaxation. The level of weighting depends on various combinations and the order of radiofrequency (RF) pulses and gradients (small linear magnetic fields superposed to static magnetic field used for slice selection, spatial encoding etc.) that create the MR imaging sequences.^30^ The simultaneous application of RF pulses and synchronized changes in the gradients lead to signal acquisition from different places in space. There are basically two types of MR imaging sequences: (i) the spin echo forming signal by two radiofrequency pulses (the first a 90° pulse, the second 180° pulse)^31,32^ and (ii) gradient echo sequence forming signal by one radiofrequency pulse (usually 5–90° pulse) and gradient reversal.^33,34^ Many parameters characterize the MR imaging sequence. The most important parameters for contrast in MR images are repetition time (TR), echo time (TE), and flip angle of excitation radiofrequency pulse. The sequence parameters should be optimized according to type of tissue and used contrast agents (their relaxation times). It should be noted that in any weighted MR image, there are always contributions from both types of relaxation. A scheme of a typical MRI system with the discussed components is illustrated in Figure 2.

### 2.4 General Requirements for MRI Contrast Agents

**Figure 3:**
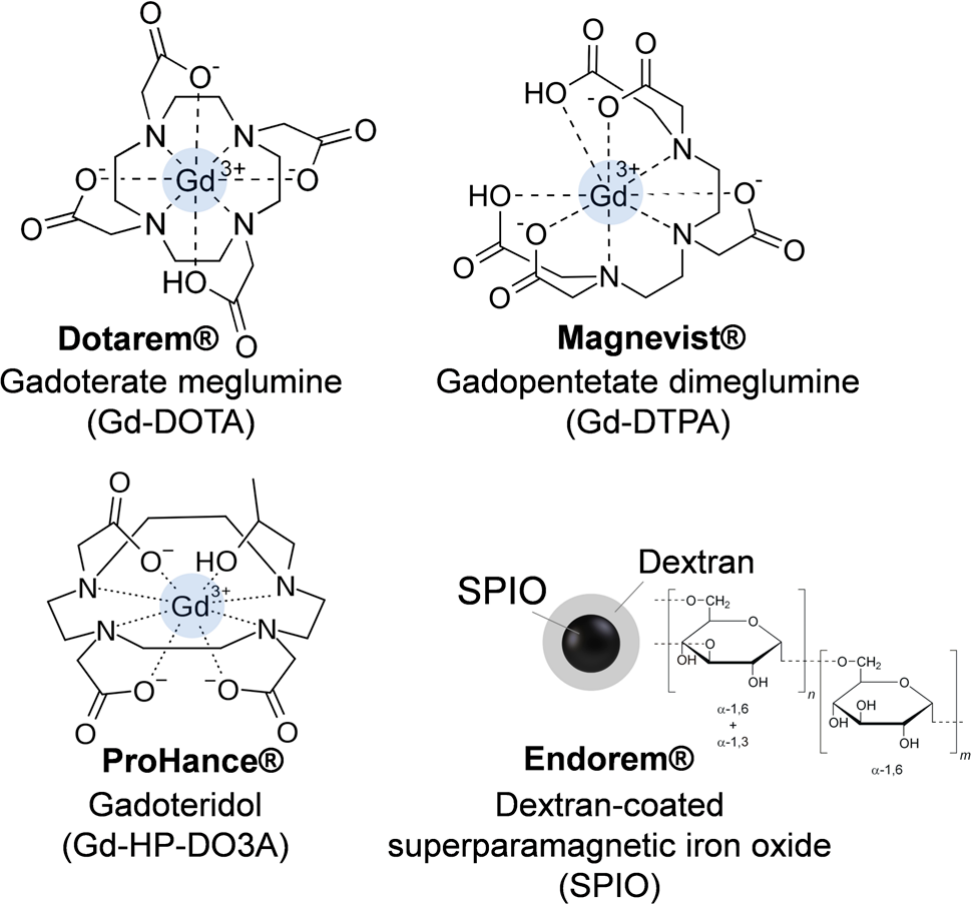
Examples of structures of commercially available contrast agents. DOTA = 2,2′,2″,2‴-(1,4,7,10-tetraazacyclododecane-1,4,7,10-tetrayl)tetraacetic acid; DTPA = 2,2′,2′′,2′′′-{[(carboxymethyl)azanediyl]bis(ethane-2,1-diylnitrilo)}tetraacetic acid; HP-DO3A= 2,2′,2″-[10-(2-hydroxypropyl)-1,4,7,10-tetraazacyclododecane-1,4,7-triyl]triacetic acid.

Contrast agents^3−7^ are used in clinical practice to improve the quality of images and to enhance detectability of pathological processes and distinguishing pathologies from healthy tissue. In experimental medicine, contrast agents are also used for a visualization of transplanted cells or imaging labels for monitoring drug delivery systems.^5,35−39^ The main purpose of contrast agents is to change a contrast in the images. In case of magnetic resonance, the change of the contrast enhancement is based on the altering of relaxation times, which goes beyond the intrinsic relaxation behavior of a targeted area such as cells or organs. The majority of MR contrast agents are either paramagnetic gadolinium ion complexes or superparamagnetic magnetite nanoparticles (Figure 3).^3−7^ These agents shorten both T_1_ and T_2_/T_2_* the relaxation times.^40−42^ This shortening, which reflects the efficiency of contrast agent, depends on many parameters including the concentration of the contrast agent. To compare the efficacy of contrast agents properly, relaxivity is used, which reflects how the relaxation rates (the inverse of the relaxation times) of the solution change depends on the concentration. Relaxivities r_1_ and r_2_, which should be as high as possible, depend on the temperature, field strength and substance in which the contrast agent is dissolved. Typical values for clinically approved contrast agents are up to 10 L· mmol^–1^·s^–1^.^43^ The relaxivities of experimental contrast agents can reach much higher values, especially for r_2_.^44,45^ The resulting r_2_/r_1_ ratio indicates whether the application of contrast will be more effective as a positive (T_1_) or negative (T_2_) contrast agent.^46^

Contrast agents are exposed to different conditions in *in vivo* experiments, so their chemical stability is a very important parameter. In general, maximum stability is required, but in some cases, under certain conditions, such as a change in pH or temperature, a stimuli-triggered degradation of the probe might be desired, for instance, to monitor a drug release.^36,47,48^

Toxicity is closely related to the chemical stability. In clinical practice, the most common Gd-based extracellular contrast agents are all chelates containing Gd(III) ions. Free gadolinium ions are highly toxic and can cause various negative side effects, such as nephrogenic systemic fibrosis, enzyme inhibition, calcium channel blockade, etc.^49−51^ Therefore, it is crucial that Gd(III) is tightly bound to the chelate to prevent its toxic effects. However, despite the Gd-chelation, there have been controversies over the agents’ safety.^52^ These concerns pointed to the importance of reducing the subject’s exposure to the contrast agent and to the importance of evaluating the clearance of administered contrast agents.

Iron-based probes are generally considered nontoxic because iron nanoparticles can be degraded and utilized by cells via the physical pathway of iron metabolism.^53^ However, some studies have shown that a high iron load in cells is toxic to the cells and can impaired their normal function.^54^ The agent toxicity is affected by many factors including size, charge and surface chemistry, etc.^55−57^ The side effects are mainly due to whether the nanoparticles undergo biodegradation in the cellular environment and what cellular reactions the degraded nanoparticles elicit.

In summary, an optimal probe for MR should possess: (i) an adequate solubility or dispersibility in water/body fluids, (ii) high relaxivities r_1_, r_2_, (iii) chemical stability, (iv) nontoxicity and reliable pharmacokinetic and pharmacodynamic properties, including biodegradability and circulation time, (v) easily modifiable surface (e.g., for a targeting ligand attachment), and possibly also (vi) a responsivity to stimuli.

## 3 MOFs as Contrast Agents in MRI

**Figure 4:**
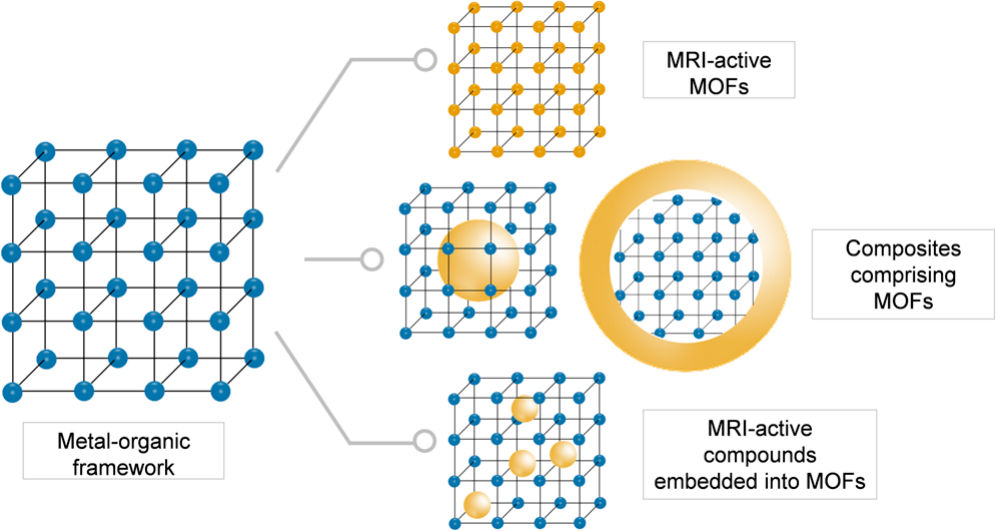
Scheme of different design strategies for preparing MRI-active materials based on MOFs; the MRI-active component is shown in a yellow color.

MRI has been considered as the key potential application of MOFs in diagnostics.^25−28^ Due to the huge design variety of MOFs,^8−10^ several different strategies for preparing MRI contrast agents based on MOFs have been reported. As the first candidates, MOFs comprising metal ions with suitable MR-properties (Table 1), namely Gd(III), Mn(II) and Fe(III), have been investigated (section 3.1 and Table 2). They offer the advantage that the MOF itself is the active component, which leads to a high efficiency due to the high metal content. Another approach, which have been reported, is an integration of MRI-active metal oxide nanoparticles, such as Fe_2_O_3_ and Fe_3_O_4_ nanoparticles, into MOFs (section 3.2 and Table 3). Last but not least, also possibilities of including contrast agents based on metal complexes into MOF pores have been proposed (section 3.3). All these three design strategies (Figure 4) are included in this review and discussed in the following chapters. As shown on many examples (Table 2 and 3), MOFs are highly promising materials for developing contrast agents in MRI. However, MOF stability (combined with a possible metal leakage) and toxicity are of a concern. Thus, it is highly important that the material properties such as the material chemical stability (in biological conditions) and toxicity (including the MOF building components) are investigated and reported.

**Table 1:** Outer Orbital, Spin, and Calculated Effective Magnetic Moment (μ_eff_) of Selected Metal Ions

metal ion	orbital	spin	μ_eff_
Gd(III)	4f^7^	7/2	7.94
Mn(II)	3d^5^	5/2	5.92
Fe(II)	3d^6^	2	4.90
Fe(III)	3d^5^	5/2	5.92

**Table 2:**
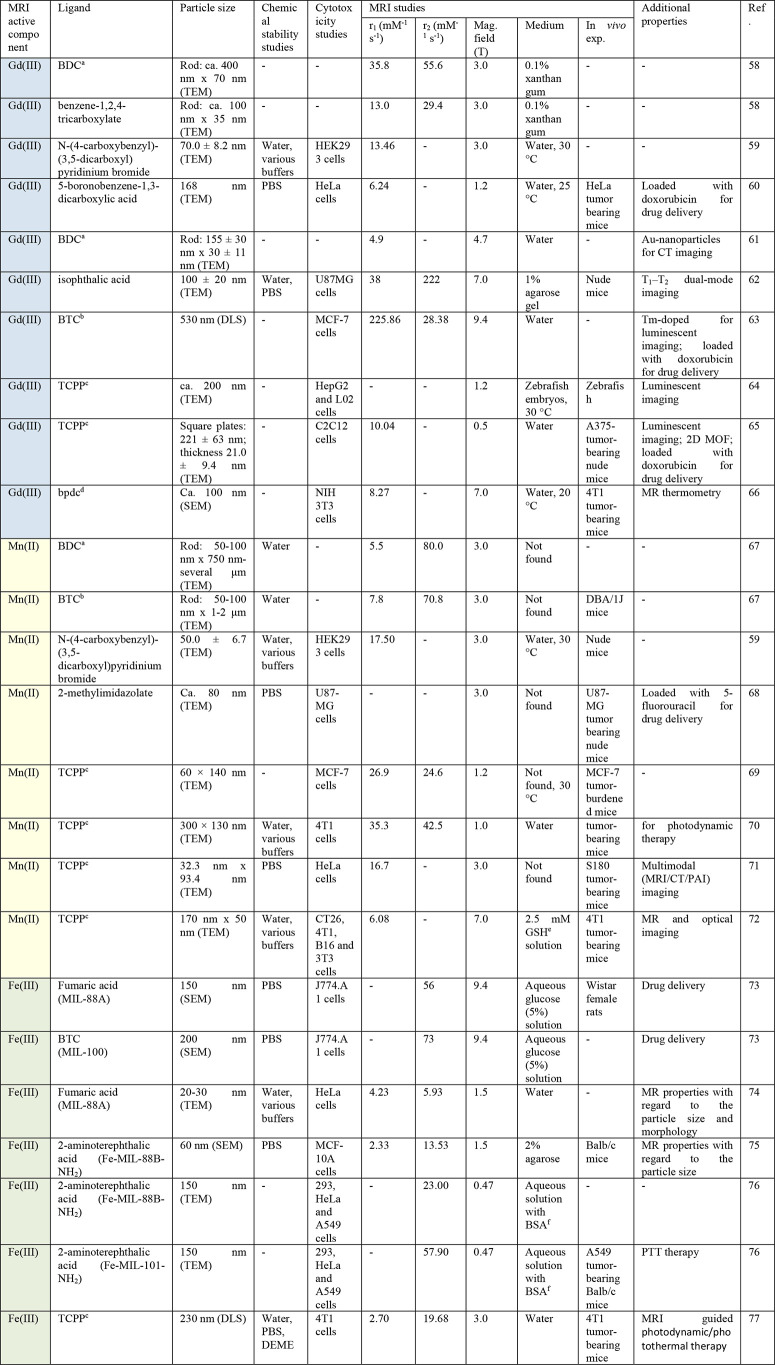

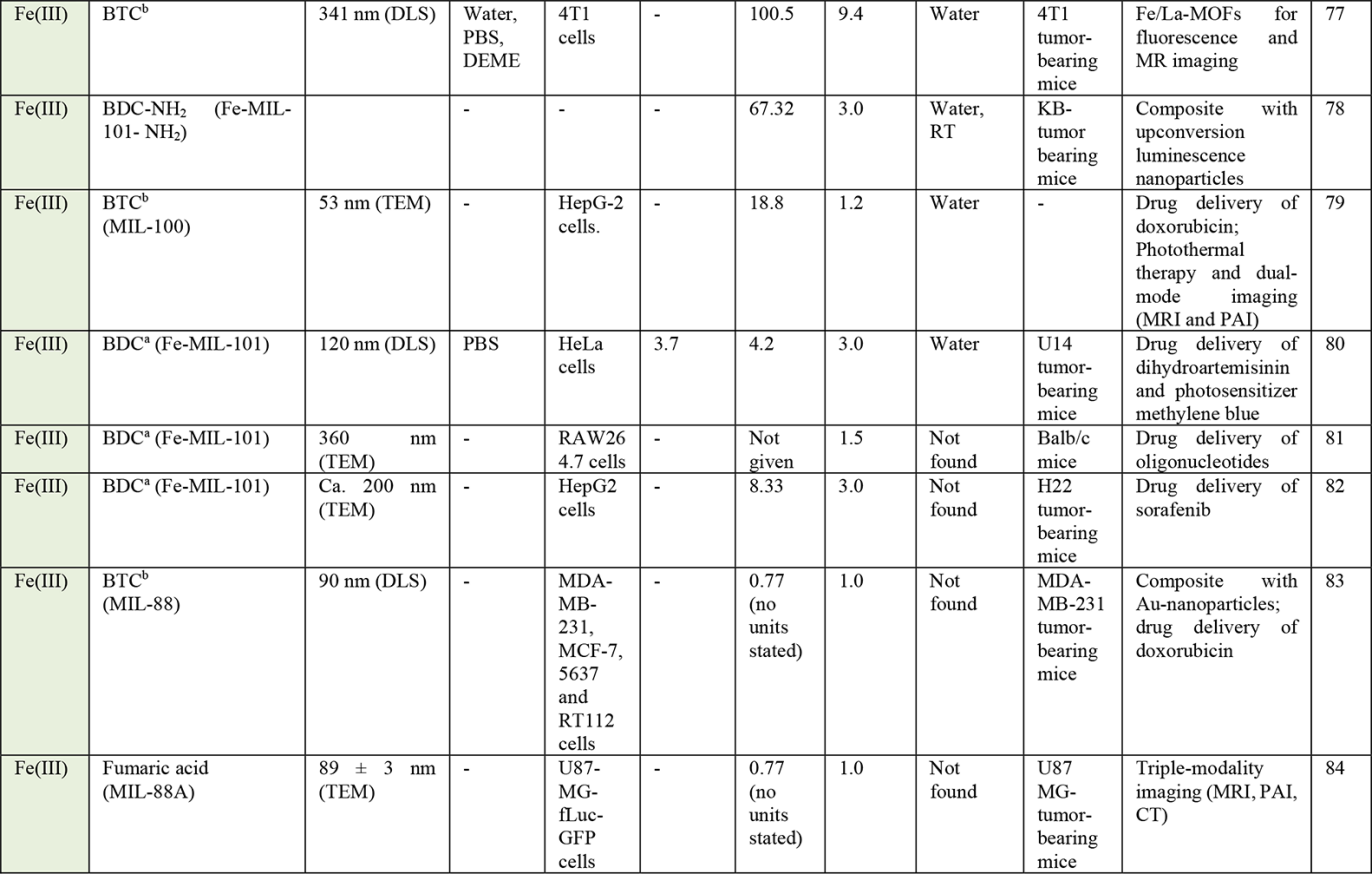
Overview of MRI-Active MOFs and Their Properties

a BDC = benzene-1,4-dicarboxylate.

b BTC = benzene-1,3,5-tricarboxylate.

c TCPP = 5,10,15,20-tetrakis(4-carboxyphenyl)-21H,23H-porphyrin.

d bpdc = 2,20-bipyridine-6,60-dicarboxylate.

e Glutathione.

f Bovine serum albumin.

**Table 3:**
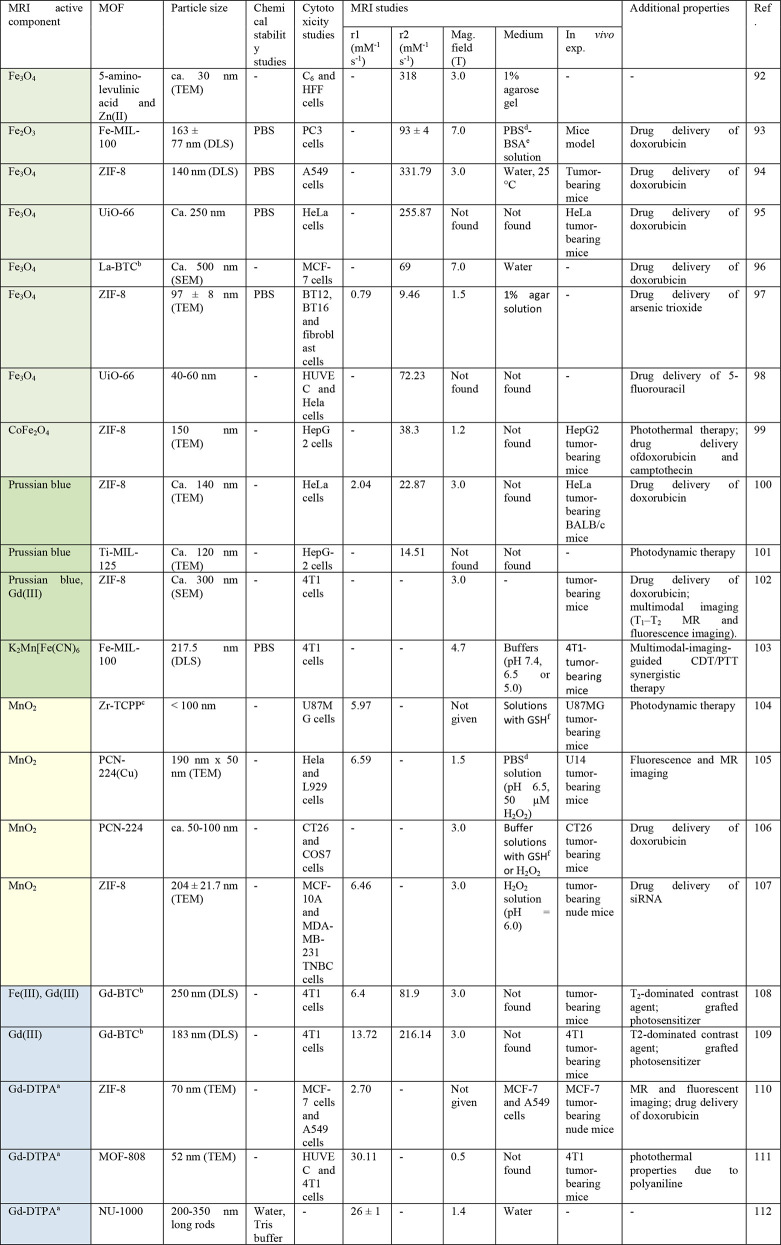
Overview of Composites Based on MOFs and MRI-Active Nanoparticles, and Their Properties

a DTPA = diethylenetriamine pentaacetic acid.

b BTC = benzene-1,3,5-tricarboxylate.

c TCPP = 5,10,15,20-tetrakis(4-carboxyphenyl)-21H,23H-porphyrin.

d Phosphate-buffered saline.

e Bovine serum albumin.

f Glutathione.

### 3.1 MRI-Active MOFs

MOFs are built up from metal ions (or clusters) and bridging organic ligands. Therefore, if metal ions with suitable magnetic properties are used, an MRI-active MOF can be prepared. Due to the magnetic properties (resulting from the number of unpaired electrons), Gd(III)-, Mn(II)- and Fe(III)-based MOFs are the most suitable (Table 1). From these, due to the concerns induced by material (in)stability and possible metal leakage, MOFs comprising Fe(III) ions (i.e., an essential metal element) are considered as the most promising. However, all three groups (Gd-, Mn- and Fe-based MOFs) have been intensively investigated over the past few years (Table 2).

#### 3.1.1 Gd-MOFs

Already in 2006, Lin at al. reported on the first Gd(III)-MOFs as contrast agents for MRI.^58^ Since then, several other Gd(III)-MOFs comprising mainly carboxylate ligands have been reported (Table 2), including ligands such as benzene-1,4-dicarboxylate^58,61^ and benzene-1,3,5-tricarboxylate,^63^ but also N-(4-carboxybenzyl)-(3,5-dicarboxyl)pyridinium bromide^59^ or 5-boronobenzene-1,3-dicarboxylate.^60^ Moreover, it has been shown that the particle size^85^ as well as the particle morphology^63^ influenced the material relaxivities, and thus these parameters could be used as efficient tools to tailor the material properties for MRI. For instance, relaxation properties of Gd(III)-MOFs comprising either benzene-1,4-dicarboxylate or benzene-1,2,4-tricarboxylate ligands were studied with regard to the crystal size.^85^ The results clearly indicated a positive correlation between the surface areas of the Gd-MOF nanoparticles with the longitudinal relaxivity in MRI. In particular, Gd-MOF nanoparticles with an average size of 82 nm yielded a high longitudinal relaxivity value of 83.9 mM^–1^ s^–1^.

**Figure 5:**
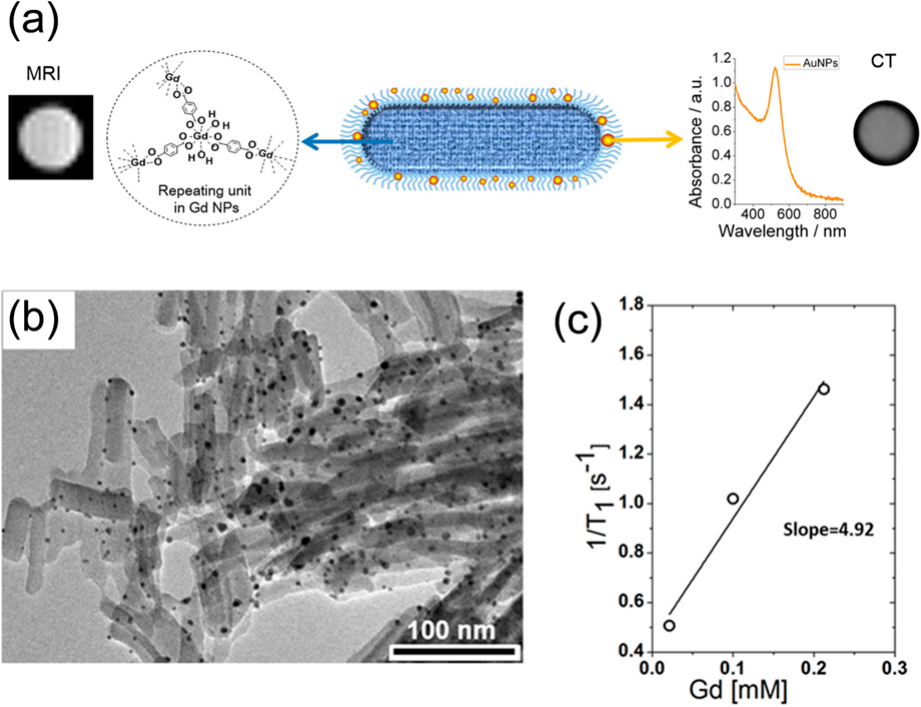
(a) Schematic representation of a Gd-MOF–Au nanostructure proposed as an agent for dual imaging. (b) TEM micrograph of the Gd-MOF–Au nanostructure and its (c) relaxation rate (1/*T*_1_) as a function of the Gd-concentration. Adapted with permission from ref (61). Copyright 2015, American Chemical Society.

Following a general trend centered around multifunctional materials, the research focus of the field of Gd-MOFs for MRI has slightly shifted over the past few years. Instead of preparing new Gd-MOFs, more attention has been paid to combining known Gd-MOFs with other materials in order to prepare agents for multimodal imaging or theranostic agents (i.e., agents combining therapy and diagnosis). For example, Icten et al. proposed to combine Gd(III)-MOFs (comprising either benzene-1,4-dicarboxylate or benzene-1,3,5-tricarboxylate ligands) with boron-10 isotope to prepare dual agents for MR imaging and neutron capture therapy.^86^ Boyes et al. combined a Gd(III)-MOF comprising benzene-1,4-dicarboxylate ligands with Au-nanoparticles (Figure 5).^61^ Au-nanoparticles, due their high atomic number and superior absorption coefficient, have been suggested as contrast agents for CT imaging.^87^ Thus, if combined with Gd-MOFs, agents for dual imaging can be obtained. The r_1_ value of the nanocomposite was 4.9 mM^–1^ s^–1^ (at 4.7 T). Meanwhile, the nanocomposite also enhanced the contrast of CT imaging, even when the Au concentration was as low as 1.66 mg/mL.

Xie et al. reported on a Eu/Gd-MOF (comprising isophthalate ligands) as a T_1_–T_2_ dual-mode contrast agents.^62^ To improve the material stability, the particle surface was coated with a layer of silica. The nanoparticles exhibited high longitudinal (38 mM^–1^ s^–1^) and transversal (222 mM^–1^ s^–1^) relaxivities (at 7.0 T). The authors speculated that such high relaxivity values were due to the rigid confinement of Gd(III)-ions in the nanosystem and slow interexchange of Gd(III) with water molecules. In another work, a Tm/Gd-MOF comprising benzene-1,3,5-tricarboxylate ligands were designed in order to prepare luminescent and MRI active nanoparticles for drug delivery.^63^ By varying the reaction parameters, different morphologies and sizes were obtained. The particles were loaded with doxorubicin as a model drug, and their surface was modified by mesoporous silica and folic acid. MRI measurements revealed an unusually high longitudinal relaxivity of 225.86 mM^–1^ s^–1^ (at 9.4 T). Li et al. suggested a Gd(III)-porphyrin MOF for magnetic resonance and fluorescence imaging due to the gadolinium and porphyrin properties, respectively.^64^ MOF nanoparticles coated with folic acid featured low biotoxicity, emitted bright red fluorescence and their MR properties were studied on zebrafish embryos and zebrafish. Similarly, Yan et al. also reported on a Gd(III)-porphyrin MOF for MR and fluorescence imaging.^65^ The prepared nanoparticles were loaded with doxorubicin as a model drug and their MRI properties were investigated both *in vitro* and *in vivo*. The r_1_ relaxivity was determined to be 10.04 mM^–1^ s^–1^ (at 0.5 T).

Gd(III)-based MOFs have been also suggested as contrast agents for magnetic resonance thermometry.^66^ Magnetic resonance thermometry is a noninvasive method which offers high spatial and temporal resolution for monitoring of temperature, for example, during cancer treatment.^88^ However, it suffers from low temperature sensitivity and image contrast. Therefore, different compounds have been developed and investigated as agents for improving the contrast. For instance, Zhang et al. prepared a Gd(III) zeolite-like MOF based on 2,20-bipyridine-6,60-dicarboxylate ligands.^66^ The nanoparticles were shown to be biocompatible and exhibited benchmark performance with respect to *in vitro* and *in vivo* MR thermal mapping. Their r_1_ relaxivity was found to be 8.27 mM^–1^ s^–1^ per Gd(III) ion (at 7.0 T). Moreover, a T_1_-based thermal map (20–50 °C) showed that the Gd-MOF was sufficiently sensitive to quantify temperature changes using T_1_ effects; even at very low concentrations (as low as 33 mM). Moreover, the high sensitivity was proven also *in vivo* on an example of thermal mapping of tumor-bearing mice.

#### 3.1.2 Mn-MOFs

Due to suitable magnetic properties, Mn(II)-ions have often been used to prepare contrast agents based on MOFs for MR imaging (Table 2). They can be either integrated as the inorganic unit to build up the MOF, or they can be included as a part of the ligand such as a coordination in porphyrins. Moreover, in the past few years, there has been a significant increase in reports of MOFs based on Mn(III)-ions as GSH-activated T_1_ contrast agents for cancer diagnosis.^72^

**Figure 6:**
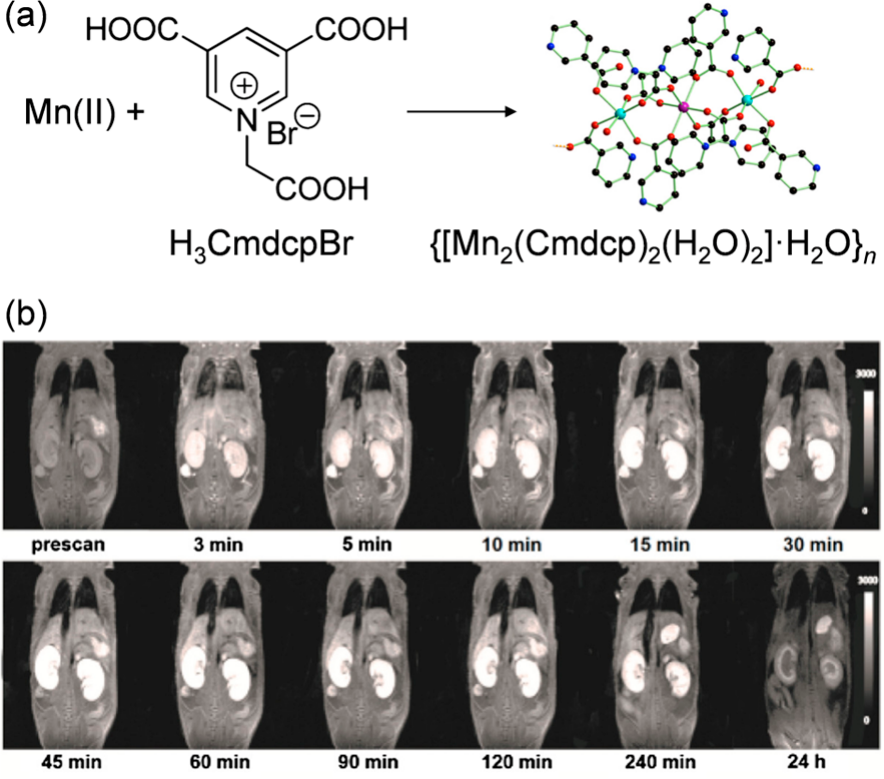
(a) Reaction scheme of the Mn(II)-MOF synthesis and (b) MR signal intensity from a dynamic study of kidneys after intravenous administration of the Mn(II)-MOF. Adapted with permission from ref (59). Copyright 2017, American Chemical Society.

The first Mn(II)-MOFs as contrast agents for MRI were reported in 2008 by Lin at al.^67^ They synthesized two Mn(II)-MOFs based on benzene-1,4-dicarboxylate and benezene-1,3,5-tricarboxylate ligands exhibiting r_1_ of 5.5 and r_2_ of 80.0 mM^–1^ s^–1^, and r_1_ of 7.8 and r_2_ of 70.8 mM^–1^ s^–1^ (at 3.0 T, per Mn ions), respectively. Since then, several other MOFs based on carboxylate ligands have been developed. For example, Chen et al. reported on a Mn(II)-MOF comprising N-(4-carboxy benzyl)-(3,5-dicarboxyl)pyridinium bromide as a ligand and studied its MR properties *in vitro* and *in vivo*.^59^ MR images of treated mice indicated that kidneys showed remarkably positive signal enhancement after 15 min with intravenous administration of the MOF and the hyperintensity of both kidneys persisted for about 240 min with no obvious tissue damage (Figure 6). The results suggested that the MOF could be used for imaging renal dysfunction, which was rather surprising considering the large particle size 50.0 ± 6.7 nm (as determined by TEM), which usually prevents a renal clearance. The authors suggested that the plausible mechanism could be that these particles disintegrated into smaller sizes through a collision and gradual decomposition over time. In another work, a MOF ZIF-8, which comprises Zn(II) ions and 2-methylimidazolate ligands, was used as a precursor to synthesize a Mn(II)-MOF suitable for visualization by MRI.^68^ By incubating ZIF-8 with Mn(II)-ions, some of the Zn(II) ions could be postsynthetically exchanged. In the final product, the ratio of Mn to Zn was 1:7. When Mn-ZIF-8 was injected intravenously into the tumor-bearing mice, enhanced T_1_-weighted MR signals could be observed at the tumor area. The signal intensity of Mn-ZIF-8 increased continuously after intravenous injection and peaked at 12 h.

Yin et al. studied a MOF comprising Zr(IV)-ions and Mn-porphyrin ligands as a T_1_-weighted MR contrast agent.^69^ The MOF exhibited a high r_1_ value of 26.9 mM^–1^ s^–1^ (at 1.2 T), therefore, it was further studied *in vivo* in a mouse model. A bright signal was detected in the liver and kidney after 1 h after the injection and decreased after 24 h. The consistent results were observed also in a MCF-7 tumor-bearing mouse model. The signal in both the liver and kidney was enhanced, but in addition to that, also the signal in the tumor was enhanced, suggesting good tumor targeting of the MOF. Moreover, *s*-nitrosothiol was conjugated to the surfaces of the MOF nanoparticles for heat-sensitive NO generation. In another work, Yang et al. reported on a MOF PCN-222(Mn), comprising Zr(IV) ions and Mn-porphyrin ligands for MRI and for photodynamic therapy.^70^ Due to the high Mn(II)-concentration through the framework, large open channels and high water affinity in the channels, a high longitudinal relaxivity of 30.3 (at 0.5 T) and 35.3 mM^–1^ s^–1^ (at 1.0 T) was measured. Moreover, the intravenous injection of the MOF into a tumor-bearing mice model provided a good T_1_-weighted contrast of the tumor area. The enhanced brightness observed in these images was maintained for approximately 8 h after injection, indicating that the MOF can provide a long-term enhanced contrast. Cheng et al. designed and studied a MOF based on hafnium clusters and porphyrin ligands functionalized with Mn(II) and coated with folic acid as a theranostic agent suitable for triple-modality imaging (MRI/CT/PAI).^71^ The r_1_ relaxivity was 16.75 mM^–1^ s^–1^ (at 3.0 T). *In vivo* MRI experiments carried out on S180 tumor-bearing mice revealed that there was an obvious enhancement in T_1_-weighted images after the injection in comparison to the preinjection images. The *in vivo* results further indicated that coating the nanoparticles with folic acid resulted in tumor targeted delivery.

MOFs comprising Mn(III)-ions can be used as responsive systems for antioxidant glutathione (GSH) as demonstrated by Zhang et al.^72^ They reported on a MOF comprising Mn(III) ions and porphyrin ligands. Interestingly, upon endocytosis by tumor cells, the MOF was decomposed into its building components (i.e., Mn-ions and free porphyrin ligands) and due to the redox reaction between Mn(III) and intracellular GSH, Mn(II)-ions were released. In *in vitro* experiments, the T_1_ signal of the MOF before and after adding GSH (2.5 mM) was recorded by MRI. Compared with a control without GSH (r_1_ of 2.65 mM^–1^ s^–1^), the T_1_-relaxation rate (r_1_) had a nearly 2.3-fold enhancement in the presence of GSH (r_1_ of 6.08 mM^–1^ s^–1^, at 7.0 T) indicating the potential of MOFs as a GSH-activated T_1_-contrast agent for cancer diagnosis. The GSH-triggered contrast enhancement was further confirmed also *in vivo*, suggesting that the nanoparticles could be used not only as contrast agents, but also to monitor MOF disintegration *in vivo*.

#### 3.1.3 Fe-MOFs

In 2010, Horcajada et al. reported on the first Fe(III)-carboxylate MOFs for MR imaging.^73^ They demonstrated that the investigated MOFs, namely MIL-88A and MIL-101, had similar transverse relaxivity (r_2_) as conventional MRI contrast agents based on iron oxides. Since then, Fe(III)-carboxylate MOFs became the most studied class of MOFs for MRI applications (Table 2).

Wuttke et al. studied the MR properties of an Fe(III)-fumarate MOF (MIL-88A) with regard to different particle morphology and size.^74^ All in all, four different variants were studied. The results showed that both r_1_ and r_2_ relaxivities tend to increase with the increase of the particle size because of higher number of paramagnetic Fe-centers in larger particles. Similarly, Khoobi et al. studied the influence of a particle size of Fe-MIL-88B (comprising Fe(III)-cluster and 2-aminoterephthalate ligands) on the MR properties.^75^ They synthesized the MOF in three different particle sizes (60, 350, and 730 nm) and determined their relaxivity. The studies revealed that by increasing the MOF crystal size, the incremental transverse relaxivity increased and the r_2_/r_1_ ratios reached values of 5.80, 42.27, and 127.00. The smallest nanoparticles were also investigated by MRI *in vivo*.

**Figure 7:**
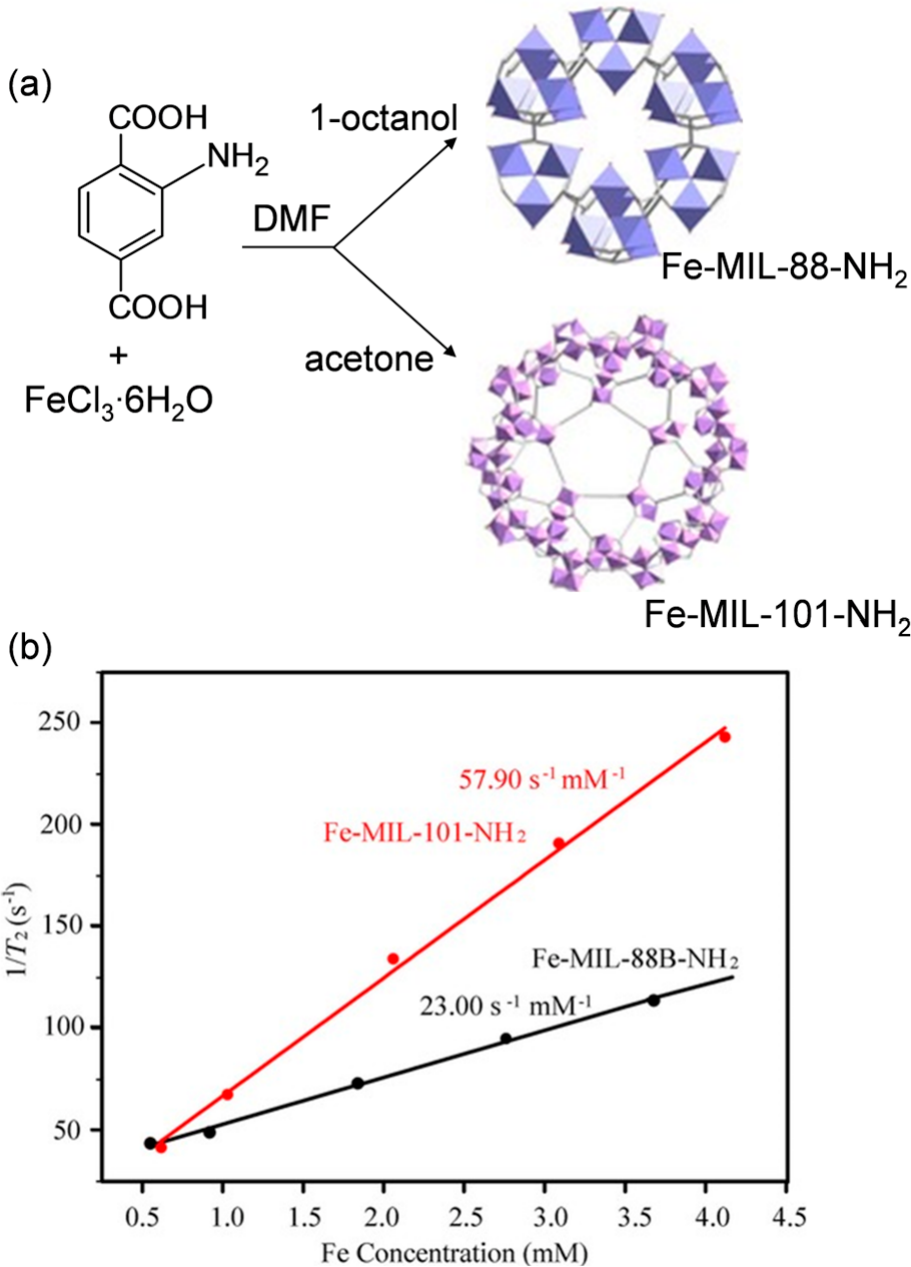
(a) Reaction scheme of the synthesis of Fe-MIL-88-NH_2_ and Fe-MIL-101-NH_2_, and (b) relaxation rate (1/T_2_) versus various Fe(III) molar concentrations for the two MOFs. Adapted with permission from ref (76). Copyright 2017, Wiley-VCH Verlag GmbH and Co. KGaA.

In another work, the MR properties of Fe-MIL-88B-NH_2_ were compared with Fe-MIL-101-NH_2_.^76^ Particles of both MOFs had an octagonal morphology and uniform size of about 150 nm. The transverse relaxivity (r_2_) value of Fe-MIL-88B-NH_2_ was determined to be 23.00 mM^–1^ s^–1^ (at 0.47 T), which was approximately 2.5-times less than the relaxivity of Fe-MIL-101-NH_2_, which was measured to be 57.90 mM^–1^ s^–1^ (at 0.47 T, Figure 7). The results suggested that the Fe-MIL-101-NH_2_ was a better candidate than Fe-MIL-88B-NH_2_ as a contrast agent in MRI, even though both materials had the same composition, morphology and size. The authors suggested that the difference in the r_2_ values could be attributed to the different interconnection of the pores resulting in a different diffusivity and exchange of water molecules within the pores. Fe-MIL-101-NH_2_ was further functionalized with graphene oxide nanosheets and studied as a material for photothermal therapy (PTT).^76^ Similarly Ren et al. reported on an Fe(III)-MOF based on porphyrin ligands as a promising platform for MR imaging and photodynamic/photothermal therapy.^77^

Wang et al. investigate heterometallic Fe/La-MOFs for fluorescence and MR imaging.^89^ The surface of these particles was coated with a layer of NH_2_-modified silica and their MR properties were investigated. The T_2_-weighted MR images showed a clear concentration-dependent contrast enhancement and the relaxivity r_2_ was determined to be 100.5 mM^–1^ s^–1^ (at 9.4 T). The author suggested that this exceptionally high value could be attributed to the seven empty 4f orbitals of La(III), which interact strongly with water molecules. In addition, water molecules are readily accessible to the paramagnetic iron atoms within the Fe/La framework through the polar amino-modified silica shell. Tang et al. reported on core–shell nanoparticles for MR and luminescence imaging prepared by combining upconversion luminescence nanoparticles and Fe-MIL-101-NH_2_.^78^ The surface of the particles was modified by a derivative of polyethylene glycol and by folic acid. The T_2_-relaxation time of water protons was shortened from about 2047 to 5.6 ms and the relaxivity r_2_ was determined to be 67.32 mM^–1^ s^–1^ (at 3.0 T). The particles were tested *in vivo* for multimodal imaging in KB-tumor bearing mice. After 24 h, the signal intensity at the tumor area decreased by about 35% indicating that the nanoparticles were successfully delivered to the tumors. In another work Wang et al. studied core–shell nanoparticles comprising a polypyrrole core and a Fe-MIL-100 shell.^79^ Due to the polypyrrole core, the prepared nanoparticles exhibited a strong absorption in the near-infrared region and possessed a good photothermal efficiency. Due to the Fe(III)-MOF shell, the particles could be detected by MRI and their relaxivity value was determined to be r_2_ = 18.8 mM^–1^ s^–1^ (at 1.2 T). Therefore, the nanoparticles were proposed as agents for photothermal therapy and multimodal imaging (MRI and PAI). Moreover, they were also tested for drug delivery of doxorubicin.

**Figure 8:**
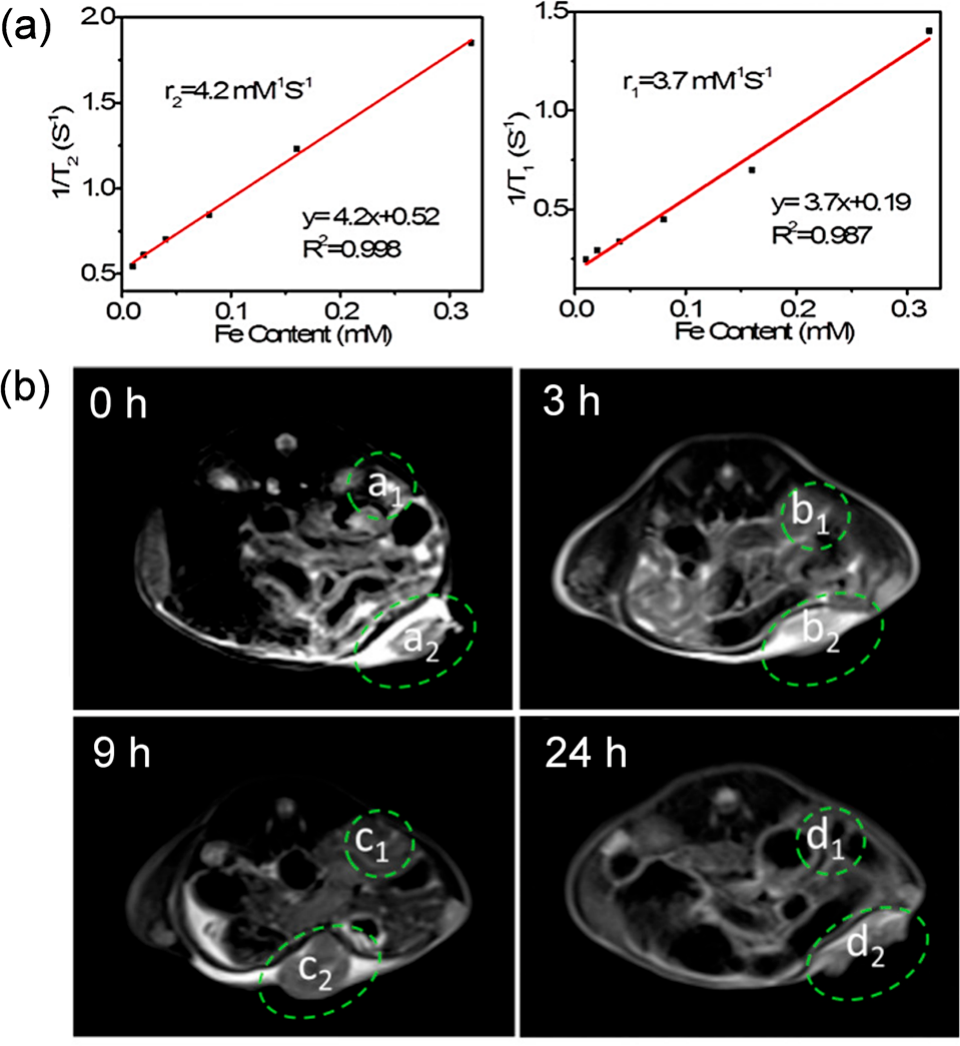
(a) Transverse (1/T_2_) (left) and longitudinal (1/T_1_) relaxation rate of suspensions of Fe-MIL-101 plotted versus the Fe-content and (b) *in vivo* MR images of a mouse bearing implanted U14 cancer after the intravenous injection of MOFs (10 μg Fe per g) at 0 h, 3, 9, and 24 h (a_1_, b_1_, c_1_, and d_1_ represent the normal tissues areas; a_2_, b_2_, c_2_, and d_2_ represent the tumor areas). Adapted with permission from ref (80). Copyright 2019, American Chemical Society.

Due to the material low toxicity, Fe(III)-MOFs are popularly used in drug delivery systems.^90^ When combined with MR imaging, theranostics (i.e., agents combining therapy and diagnosis within one system) are prepared. For instance, Fe-MIL-101 was reported as a theranostic agent for MRI and drug delivery of an anticancer drug dihydroartemisinin and photosensitizer methylene blue.^80^ The MOF nanoparticles were coated with polylactic acid and polyethylene glycol to achieve controllable drug release and good biocompatibility. The transverse relaxivity r_2_ was determined to be 4.2 mM^–1^ s^–1^, while the longitudinal relaxivity r_1_ was only 3.7 mM^–1^ s^–1^ (at 3.0 T), suggesting that the nanoparticles could be used as a T_2_ contrast agent (Figure 8). *In vivo* T_2_ weighted MR images indicated an effective enrichment of the nanoparticles within a tumor area. The MR signal of the tumor areas was much stronger after 9 h and continued up to 24 h postinjection. Qu et al. reported on Fe-MIl-101 for drug delivery of unmethylated cytosine–phosphate–guanine oligonucleotides for enhancing an immune response and MR imaging.^81^ Both *in vitro* and *in vivo* MRI studies were carried out. Similarly, Xu et al. reported on Fe-MIL-101 for drug delivery of sorafenib.^82^ To enhance the targeting ability, the nanoparticle surface was functionalized with an iRGD peptide. The transverse relaxivity r_2_ was determined to be 8.33 mM^–1^ s^–1^ (at 3.0 T). Kulinowski et al. reported on Fe-MIL-101-NH_2_ for drug delivery of isoniazid and examined the nanoparticle MR properties on a lung tissue phantom and on rat lungs *ex vivo*.^91^

Fe(III)-MOFs can be also combined with gold nanoparticles to prepare theranostics for photothermal therapy. Whereas the Fe(III)-MOF is used for drug loading and MR imaging, Au-nanoparticles, when irradiated with a laser, can be used as agents for photothermal therapy and heat induced drug release. Tian et al. combined Au-nanoparticles with a MOF based on Fe(III) ions and benzene-1,3,5-tricarboxylate ligands (MIL-88).^83^ The r_2_ relaxivity of the agent was determined to be 0.77 and its feasibility in *in vivo* MR imaging was investigated. The particles were injected into MDA-MB-231 tumor-bearing mice and time-dependent T_2_ MR imaging was carried out. The signal intensity increased 1 h postinjection and continued to rise over the next 24 h. In another work, the authors combined Au-nanoparticles and MIL-88A to prepare core–shell nanoparticles for multimodal imaging.^84^ The Au-core possessed CT enhancement and PAI optical properties, while the MOF shell exhibited a *T*_2_-weighted MR property. The surface of the nanoparticles was modified by poly(ethylene glycol)-carboxylic acid to improve the dispersibility of the particles. In order to investigate the nanoparticles as a platform for MRI of tumors, T_2_-weighted images were obtained from mice with U87MG tumors. A remarkable darkening effect was observed in the tumors of injected mice after 12 h suggesting a high passive uptake of the nanoparticles by tumors.

### 3.2 Composites Based on MOFs and MRI-Active Nanoparticles

**Figure 9:**
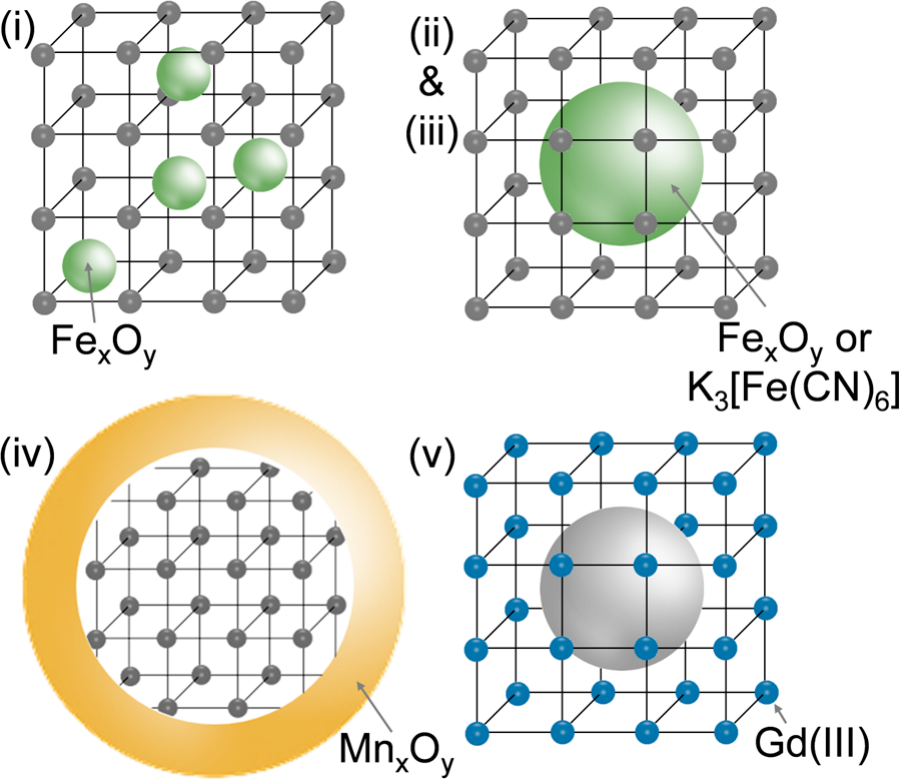
Different structures of composites based on MOFs studied as potential MRI contrast agents: (i) iron oxide nanoparticles in MOF matrices, (ii) core–shell iron oxide-MOF particles, (iii) core–shell Prussian blue-MOF nanoparticles, (iv) manganese oxide MOF nanoparticles, and (v) Gd-MOFs with a core–shell structure.

Besides MOFs based on MRI active metal ions described in the previous section, a strategy of including MRI-active nanoparticles into MOFs has been extensively studied over the past few years (Table 3). For the purpose of this review, we divided these materials into five groups (Figure 9): (i) iron oxide nanoparticles in MOF matrices, (ii) core–shell iron oxide-MOF particles, (iii) core–shell Prussian blue-MOF nanoparticles, (iv) manganese oxide MOF nanoparticles, and (v) Gd-MOFs with a core–shell structure. By introducing MRI active nanoparticles within a MOF, the variety of MOFs, which can be used is enlarged, because these MOFs do not have to comprise MRI-active metal ions. In such approach, the MRI active component is responsible for the imaging and the porous MOF can be used for loading other active species including drug molecules. Such materials could be useful for multitargeted medical applications in both diagnosis and therapy.

#### 3.2.1 Iron Oxide Nanoparticles in MOF Matrices

**Figure 10:**
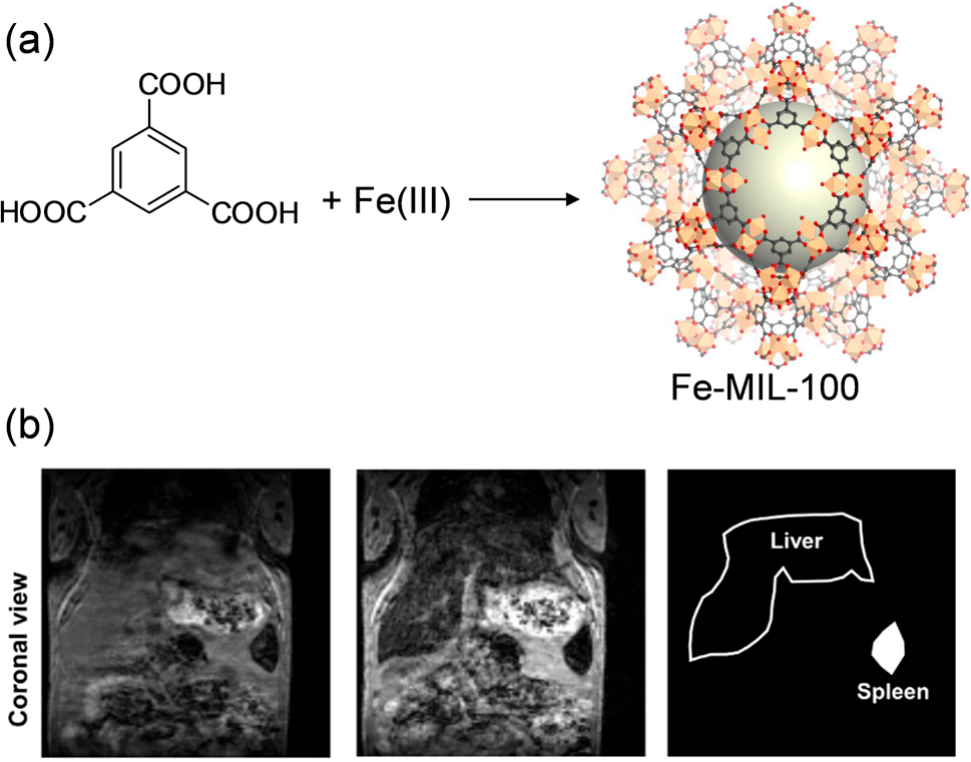
(a) Reaction scheme of the synthesis of Fe-MIL-100 and (b) 3D T_2_*-weighted gradient echo images of the mouse abdomen before and after administration of the Fe-MIL-100/Fe_2_O_3_ material showing a decrease in the liver and spleen signal upon the nanoparticle administration. Adapted with permission from ref (93). Copyright 2017, Elsevier.

Iron oxide nanoparticles can be embedded into a MOF matrix either during the synthesis or postsynthetically. For instance, Khoobi et al. first prepared Fe_3_O_4_ nanoparticles, which were then integrated within a MOF constructed from 5-aminolevulinic acid and zinc(II) ions during the MOF synthesis.^92^ The resulted particles were about 30 nm large and exhibited a high transverse relaxivity of 318 mM^–1^ s^–1^ (at 3.0 T). In another work, Steuneu et al. synthesized separately MOF nanoparticles of Fe-MIL-100 and citrate coated Fe_2_O_3_ nanoparticles (7 ± 3 nm large).^93^ By combining aqueous solutions of the MOF and Fe_2_O_3_ nanoparticles at a pH value, in which both materials exhibited opposite surface charges, an efficient coupling of the agents with a fine control over the MOF/Fe_2_O_3_ ratio was achieved. At 10% w/w loading of Fe_2_O_3_, the composite had a high value r_2_ of 93 mM^–1^ s^–1^ (at 7.0 T), which was similar to the clinically approved contrast agents. Since only small amounts of Fe_2_O_3_ were needed to facilitate the efficient imaging performance, the MOFs retained their porosity after conjugation, allowing the authors to load the pores with an anticancer drug doxorubicin. Furthermore, the application of these materials as MRI contrast agents was demonstrated *in vivo*. Their high T_2_*-effect led to a homogeneous decrease in the liver and spleen signal (Figure 10), generating a 52% decrease in signal-to-noise ratio and suggesting rapid internalization of the MOF/Fe_2_O_3_ composites.

#### 3.2.2 Core–Shell Iron Oxide-MOF Particles

To synthesized core–shell iron oxide-MOF particles, first, the magnetic core is prepared and then the MOF shell is grown around it.^113^ The MOF growth can be done as a one-step process^94,97^ or stepwise by a layer-by-layer growth.^95,96,98,99^

Chen et al. reported on ZIF-8 with a core formed by carbon-encapsulated superparamagnetic Fe_3_O_4_ nanoparticles.^94^ While the carbon dots could serve as agents for fluorescent imaging, the Fe_3_O_4_ nanoparticles were included to enable the detection by MRI. The star relaxivity (r_2_*) of the nanoparticles was determined to be 331.79 mM^–1^ s^–1^ (at 3.0 T) and the cellular uptake of the nanoparticles was studied in A549 cells by MRI. Compared with untreated cells, darker signal intensity was observed after the treatment of the cancer cells with the nanoparticles indicating that the nanoparticles could be endocytosed by the cells effectively. Moreover, *in vivo* MRI studies on tumor-bearing mice were carried out. The T_2_*-signal in liver became darker suggesting that the liver was the major organ for the metabolism and clearance of the nanocarriers.

Fu et al. reported on a Fe_3_O_4_–UiO-66 platform for delivery of doxorubicin.^95^ They synthesized Fe_3_O_4_-clusters with a diameter of 150 nm and coated them with UiO-66. By varying the reaction conditions, particles with three different thickness of the MOF shell −5, 25, and 50 nm, were prepared. T_2_-weighted MR images of Fe_3_O_4_–UiO-66 showed an expected concentration-dependent darkening effect with a high transverse relaxivity (r_2_) of 255.87 mM^–1^ s^–1^, and the r_2_ values decreased as the thickness of the UiO-66 shell increased due to the reduced ratio of Fe_3_O_4_ to UiO-66 in the composite. The feasibility of the Fe_3_O_4_–UiO-66 nanoparticles for *in vivo* MRI was tested on HeLa tumor-bearing mice. A remarkable darkening effect was observed in the tumor area just 1 h postinjection and the MR image became even darker at 9 h postinjection. In another work, a Fe_3_O_4_–La-MOF comprising benzene-1,3,5-tricarboxylate ligands was synthesized by the layer-by-layer method and used for drug delivery of doxorubicin.^96^ In the synthesis, graphene oxide (GO) was added to form MOF/GO (10%) layers. Composites comprising 10 and 20 layers were prepared. With an increase of the number of layers, the overall particle size increased to 300 nm for 10 layers and to 500 nm for the particles with 20 layers. The relaxivities r_2_ for the nanoparticles with 10 and 20 layers were determined to be 35 mM^–1^ s^–1^ and 69 mM^–1^ s^–1^ (at 7.0 T), respectively. The higher r_2_ for the nanoparticles with 20 layers was explained by the higher content of GO in these particles. The high content of hydrophilic groups on the GO resulted in enhanced accessibility of water to the magnetic core.

The average diameter of the majority of reported Fe_3_O_4_-MOF core–shell nanoparticles is over 200 nm,^113^ which is not optimal for drug delivery applications in cancer treatment, because it has been suggested that only small nanoparticles (below 150 nm) can be accumulated in tumors via the enhanced permeability and retention effect.^114^ Therefore, we recently reported on the synthesis of Fe_3_O_4_-MOF core–shell nanoparticles below 100 nm large (97 ± 8 nm as determined by TEM).^97^ To prepare such small nanoparticles, it was necessary to synthesize very small Fe_3_O_4_-clusters (here, only 35.1 ± 4.5 nm large), which were then coated with a MOF, in this work with ZIF-8. The nanocomposite with a r_2_/r_1_ ratio of 12.39 was loaded with arsenic trioxide (a promising anticancer drug^115^) and studied as a potential theragnostic agent. Similarly, Yang et al. also reported on core–shell Fe_3_O_4_-MOF nanoparticles, which were only 40–60 nm large (as determined by SEM).^98^ The nanoparticles based on Fe_3_O_4_ and a MOF UiO-66 were proposed for MRI and drug delivery applications. To gain a control over the drug release, the particle surface was functionalized by pillararene-based pseudorotaxanes as tightness-adjustable nanovalves. The particles were loaded with 5-fluoruracil as a model drug and their MR properties were investigated.

Yin et al. reported on CoFe_2_O_4_-ZIF-8 nanoparticles for MRI and photothermal therapy.^99^ A mesoporous CoFe_2_O_4_-core was included to act as a T_2_-weighted MRI agent, PTT agent and a platform for loading doxorubicin. To prevent premature drug release, the core was coated by polydopamine which also facilitated the coating with ZIF-8. The ZIF-8 shell served for loading camptothecin (CPT) and enabled a pH-responsive drug release.

#### 3.2.3 Prussian Blue Nanoparticle in MOF Matrices

Prussian blue, K_3_[Fe(CN)_6_], consists of Fe ions connected by CN^–^ anions. Due to its unique Fe(II)–C≡N–Fe(III) structure [Fe(II): low spin, S = 0; Fe(III): high spin, S = 5/2), Prussian blue nanoparticles can serve both T_1_ and T_2_ MRI contrast agents.^116^ Recently, nanoparticles of Prussian blue has been combined with MOFs to prepare theranostic agents. For instance, Chen et al. coated Prussian blue nanoparticles with a ZIF-8 shell and used the nanocomposite for delivery of doxorubicin.^100^*In vitro*, the r_1_ and r_2_ values were measured to be 2.04 mM^–1^ s^–1^ and 22.87 mM^–1^ s^–1^ (at 3.0 T), respectively. Subsequently, *in vivo* MR images were also conducted. Compared with the preinjected images, both T_1_-weighted and T_2_*-weighted images of a tumor site exhibited enhanced MR signal at 24 h postinjection indicating an accumulation of the nanocomposite in the tumor. The MR signal of liver became darker suggesting that liver was the major organ for the metabolism and clearance of the nanoparticles. In another work, nanoparticles of Prussian blue were coated with a MOF Ti-MIL-125.^101^ While the core serves as a contrast agent for MRI, the outer shell of Ti-MIL-125 can serve as photosensitive reagent for photodynamic therapy (PDT). The particles exhibited an r_2_ value of 14.51 mM^–1^ s^–1^, which indicated that they could be used as potential T_2_ MRI contrast agents. Wang et al. reported on Prussian blue nanoparticles doped with Gd(III) and Tm(III), and subsequently coated with a MOF ZIF-8 and polydopamine to prepare composite nanoparticles suitable for drug delivery of doxorubicin and multimodal imaging (T_1_–T_2_ dual-mode MR and fluorescence imaging).^102^ A quantitative *in vivo* analysis in a mouse model confirmed that after the injection of the nanoparticles, T_1_-weighted images became brighter, while the T_2_-weighted images were darker.

By combining Prussian blue nanoparticles with MOFs, the drug loading capacity is enlarged. However, the nanoparticles of Prussian blue themselves are also porous and can be used for drug loading. For example, Tian et al. loaded nanoparticles of Prussian blue with sorafenib and investigated their MRI properties.^117^ Moreover, analogues of Prussian blue can also be used in a combination with MOFs for applications in MRI. For instance, nanoparticles of K_2_Mn[Fe(CN)_6_] coated with a MOF Fe-MIL-100 were studied as agents for multimodal imaging and synergistic therapy.^103^ The authors showed that in a mildly acidic tumor microenvironment, Mn(II) ions were released which resulted in an “ON” state of both T_1_-weighted magnetic resonance imaging and photoacoustic signals.

#### 3.2.4 Manganese Oxide MOF Nanoparticles

In comparison to the design of iron oxide-MOF composites, in which Fe_3_O_4_ usually forms the core, manganese oxide, namely MnO_2_, is usually used as a shell to coat MOF nanoparticles. However, also examples of Mn_3_O_4_ nanoparticles embedded into MOFs are known. For example, Kefayad et al. reported on manganese oxide (Mn_3_O_4_) nanoparticles conjugated with poly(acrylic acid) incorporated into a MOF ZIF-8 in order to prepare a pH-sensitive drug delivery system suitable for MR imaging.^118^ The r_1_ relaxivity of the nanoparticles was measured to be 3.3 mM^–1^ s^–1^. In another work, Zhang et al. reported on MnFe_2_O_4_-MOF core–shell nanoparticles.^119^ The MnFe_2_O_4_ nanoparticles were included because of their catalase-like and glutathione peroxidase-like activities. As a MOF, porphyrin-based MOF, which can act as a photosensitizer, was selected. Moreover, the MnFe_2_O_4_-MOF showed relaxivity r_1_ of 2.94 mM^–1^ s^–1^ and r_2_ of 51.53 mM^–1^ s^–1^ due to the paramagnetic manganese and iron ions. To evaluate the potential of the nanoparticles as contrast agents *in vivo*, T_1_ weighted MRI imaging of tumor bearing mice was performed. A positive contrast in the tumor area could be detected at 24 h after the injection indicating the nanoparticles could be used as contrast agents in MRI.

**Figure 11:**
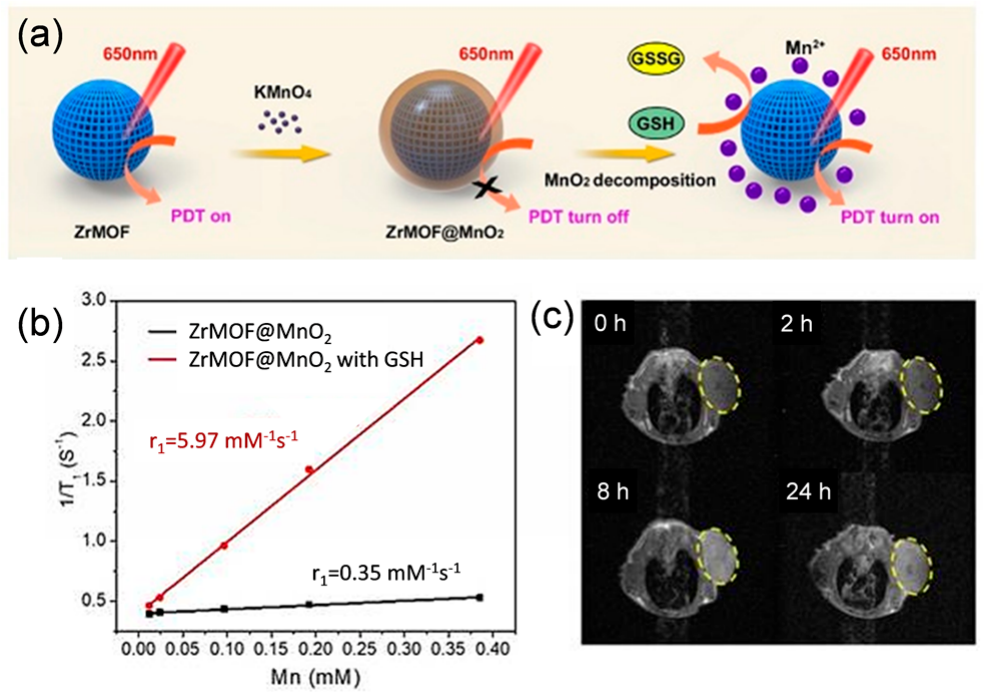
(a) Scheme of the synthesis of GSH-responsive Zr-MOF@MnO_2_ hybrid nanoparticles for MRI-guided enhanced tumor therapy, (b) longitudinal (1/T_1_) relaxation rate of suspensions of the particles without and in the presence of GSH plotted versus the Mn-content, and (c) T_1_-weighted images of a mouse at different times after intravenous injection with PEGylated Zr-MOF@MnO_2_ hybrid nanoparticles. Adapted with permission from ref (104). Copyright 2019, Theranostics.

MnO_2_, which is often used as a shell in MOF-composites, is stable at physiological conditions with only a weak T_1_ weighted MR imaging ability. However, when MnO_2_ encounters glutathione (GSH), Mn(IV)-ions are gradually reduced to Mn(II), which enhances the T_1_-MR contrast. Therefore, MnO_2_ has been suggested as a GSH sensing platform detectable by MRI.^120^ Yin et al. reported on a Zr(IV)-porphyrin MOF coated with MnO_2_ to realize an oxidation of GSH by MnO_2_ for enhanced photodynamic therapy.^121^ MOF particles were first coated with poly(allylamine hydrochloride), then a uniform MnO_2_ layer was coated on the surface by a redox reaction between KMnO_4_ and poly(allylamine hydrochloride). The MRI properties were investigated in the presence of GSH and without. In a solution with GSH, the T_1_ relaxation rate r_1_ was 6.59 mM^–1^ s^–1^, which was 7.7-fold higher than that without GSH. The nanoparticles were further tested *in vivo* in MR imaging of tumor-bearing mice. The images of mice were recorded after local injection of the nanoparticles into tumor tissues and the muscle on the opposite side. A strong T_1_-MR signal was observed at the tumor area because of the reduction of MnO_2_ to Mn(II) by GSH in tumor cells. In comparison, the muscle section showed a less T_1_-signal after the injection. Simultaneously, strong T_1_-signals were observed in the kidney due to the rapid renal excretion of Mn(II)-ions. Similarly, also Chen et al, reported on a Zr(IV)-porphyrin MOF coated with MnO_2_ as agents for photodynamic therapy (Figure 11).^104^ The relaxivity of the nanoparticles was determined to be 5.97 mM^–1^ s^–1^ in the presence of GSH. Moreover, it was shown that not only MRI-properties, but also fluorescence and photodynamic activities could be turned on by GSH. In another work, a Zr(IV)-MOF based on porphyrin ligands coated with a MnO_2_ shell was studied as an agent for bimodal imaging (fluorescence and MR imaging).^122^ The authors suggested that due to the responsiveness of the MnO_2_ layer to H^+^ and H_2_O_2_, O_2_ can be produced, which can enhance O_2_-mediated singlet oxygen (^1^O_2_) generation for photodynamic therapy. Moreover, during the redox reaction, Mn(II)-ions are released, which can act as contrast agents in MRI. As expected, the T_1_-weighted images of the nanoparticles in H_2_O_2_ solution (pH = 5.5) were much brighter than those in neutral aqueous solution and the r_1_ relaxivity in a presence of H_2_O_2_ was determined to be 4.51 mM^–1^ s^–1^, while in a neutral aqueous solution, it was only 0.03 mM^–1^ s^–1^. Similarly, Yang et al. studied nanoparticles of Zr(IV)-MOF comprising porphyrin ligands with coordinated Cu(II)-ions coated by a MnO_2_ shell as agents for fluorescence and MR imaging.^105^ The r_1_ relaxivity at pH 6.5 in the presence of H_2_O_2_ was determined to be 6.59 mM^–1^ s^–1^ (at 1.5 T). In another work, porphyrin-based MOF nanoparticles coated with a MnO_2_ shell were used for drug delivery of doxorubicin and T_1_-MRI.^106^ MR imaging of nanoparticles treated with GSH or incubated in buffer solutions with different pH values was performed. As expected, it was found that the addition of GSH significantly enhanced the signal, which confirmed the effective degradation of MnO_2_ into paramagnetic Mn(II).

Shen et al. reported on a MOF ZIF-8 coated with MnO_2_ for delivery of siRNA against pyruvate kinase muscle isozyme M2.^107^ siRNA was loaded in situ during the MOF synthesis. After that, the particles were coated with MnO_2_ and finally, molecules of folic acid as targeting ligands were attached to the particle surface. The r_1_ relaxivity of such particles was measured to be 6.46 mM^–1^ s^–1^ (at 3.0 T). This result was confirmed by *in vivo* experiment in mice receiving intravenous injection of the nanoparticles where the signal intensity in T_1_-weighted MR images rapidly increased at the tumor area at 24 h postinjection and reached a peak at 48 h postinjection.

#### 3.2.5 Gd-MOFs with a Core–Shell Structure

**Figure 12:**
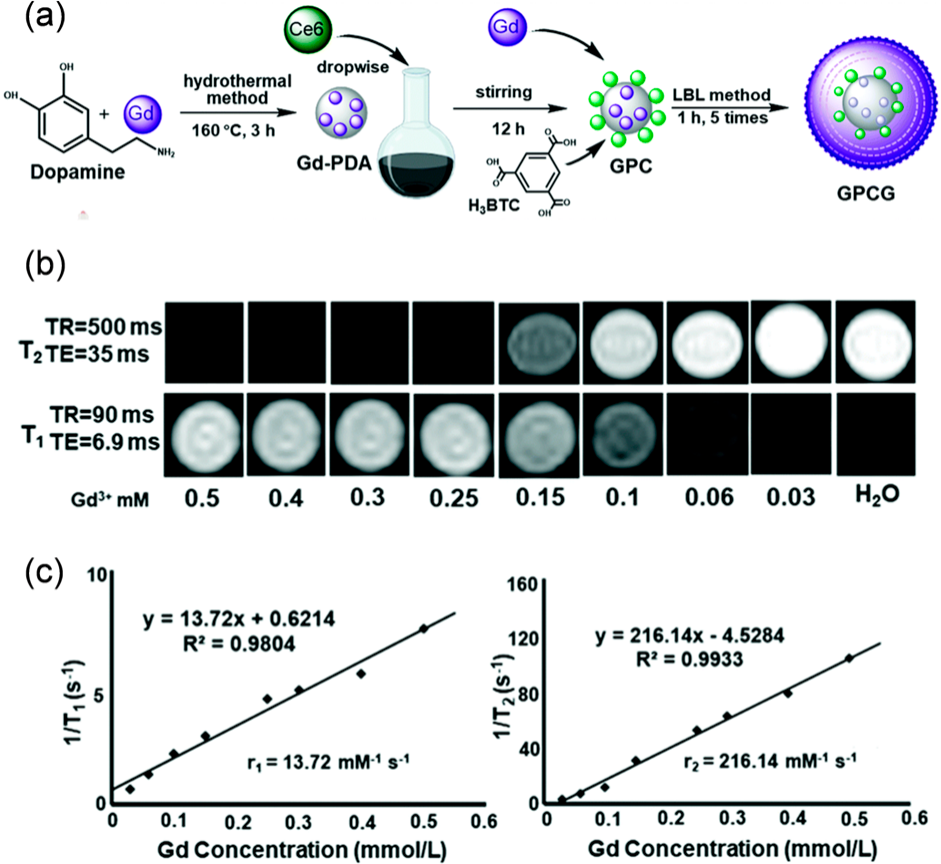
(a) Schematic synthesis of Gd-doped polydopamine Gd-MOF nanoparticles, (b) their T_1_ and T_2_ MR images at different concentrations, and (c) their relaxation rates (1/T_1,2_) versus different Gd-concentrations. Adapted with permission from ref (109). Copyright 2021, Royal Society of Chemistry.

Fan et al. reported on a composite comprising a core based of self-assembly of doxorubicin and Fe(III) ions.^108^ In a subsequent step, the core was coated by a Gd(III)-MOF based on benzene-1,3,5-tricarboxylate ligands, followed by grafting photosensitizer indocyanine green onto the surface, to enable multimodal imaging. In the design, the Gd-MOF shell did not act only as an MRI contrast agent, but also provided a protection for the core, and thus a control for the drug release. The relaxivity of the nanoparticles was determined to be r_1_ of 6.4 mM^–1^ s^–1^ and r_2_ of 81.9 mM^–1^ s^–1^ (at 3.0 T) with a high r_2_/r_1_ ratio (*>*12) indicating that the nanocomposite was a T_2_-dominated contrast agent. In another work, Fan et al. combined a Gd(III)-MOF with polydopamine.^109^ First, Gd-ions and polydopamine were combined to prepare Gd-doped polydopamine nanoparticles. Then, a widely used PDT photosensitizer chlorin e6 (Ce6) was loaded into the nanoparticles, followed by coating the nanoparticles with a Gd(III)-MOF comprising benzene-1,3,5-tricarboxylate ligands via a stepwise assembly process (Figure 12). After 5 reaction steps, the size of the nanoparticles gradually increased from 128 to 183 nm (determined by DLS). The relaxivity was determined to be r_1_ of 13.72 mM^–1^ s^–1^ and r_2_ of 216.14 mM^–1^ s^–1^ (at 3.0 T). Due to the high r_2_/r_1_ ratio (*>*15), the nanoparticles could be assigned as a T_2_-dominated contrast agent. Zhang et al. reported on integrating Gd-doped silica nanoparticles within a MOF ZIF-8, which was loaded with chlorin e6 and doxorubicin (anticancer drug) in order to prepare an agent for drug delivery, and bimodal MR and fluorescent imaging.^106^ Moreover, the particle surface was functionalized with folic acid to enable tumor targeted delivery. The relaxivity of such particles was determined to be r_1_ of 2.70 mM^–1^ s^–1^. Further, the particles were administrated via intraperitoneal injection into MCF-7 tumor-bearing nude mice. After the injection, a strong MRI signal was observed at the tumor area, indicating the ability of the magnetic resonance contrast-strengthening effect of the nanocomposites.

### 3.3 Gadolinium Complexes Embedded into MOFs

An insufficient stability, and thus uncontrolled metal leakage, of Gd-MOFs is a concern regarding their biomedical application as contrast agents in MRI. Therefore, as an alternative, introducing stable gadolinium complexes within the pores of MOFs has been suggested. For instance, Yang et al. reported on incorporating Gd(III)-complexes (Gd-DTPA, DTPA = diethylenetriamine pentaacetic acid) within the pores of MOF-808.^111^ The MOF nanoparticles were first immersed in a DTPA sodium salt aqueous solution to graft DTPA molecules onto MOF-808, and then submerged into a Gd(NO_3_)_3_ solution leading to chelation of Gd(III) by DTPA within the MOF. The surface of the particles was further modified with polyaniline. The geometric restriction of polyaniline on the surface largely ensured that Gd-DTPA remained inside the MOF pores. At the same time, the photothermal properties of polyaniline also provided a possibility for photothermal therapy. In another work, Meade et al. reported on inserting Gd-complexes (Gd-DTPA) into pores of different MOFs in order to investigate the influence of the framework structure and composition on relaxivity.^112^ The authors postsynthetically incorporate Gd-DTPA into Zr-MOFs (NU-1000 and NU-901) using solvent-assisted ligand incorporation. The impact of a particle size (nanosized vs microsized) and the MOF type on proton relaxivity was investigated. The Gd-functionalized nanoparticles of NU-1000 displayed the highest loading of the Gd(III) complex (1.9 ± 0.1 complexes per node) and exhibited the most enhanced proton relaxivity (r_1_ of 26 ± 1 mM^–1^ s^–1^ at 1.4 T).

## 4 MOFs in Heteronuclear MRI

^1^H MRI is by far the most common technique due to high ^1^H abundance and high sensitivity compared to other nuclei. However, also other nuclei can be detected, including ^19^F and ^129^Xe.

### 4.1 MOFs and Hyperpolarized ^129^Xe MRI

In ^129^Xe MRI, hyperpolarized ^129^Xe is usually used, because it can boost the signal sensitivity to over 10 000-fold compared with conventional MRI.^123^ Hyperpolarization is a process, which results in the nuclear spin polarization of a material in a magnetic field far beyond thermal equilibrium conditions determined by the Boltzmann distribution;^124^ meaning that less of the spin states cancel each other resulting in a higher sensitivity. The process of hyperpolarization is usually performed using spin-exchange optical pumping using circularly polarized light.^124^ However, the polarized light cannot directly transfer angular momentum to the gas nuclei, thus, an alkali metal atom such as rubidium is used as an intermediary. Subsequently, when ^129^Xe nuclei collide with Rb, the polarization is transferred from the Rb valence electron to the nuclear spin of the noble gas atom.

**Figure 13:**
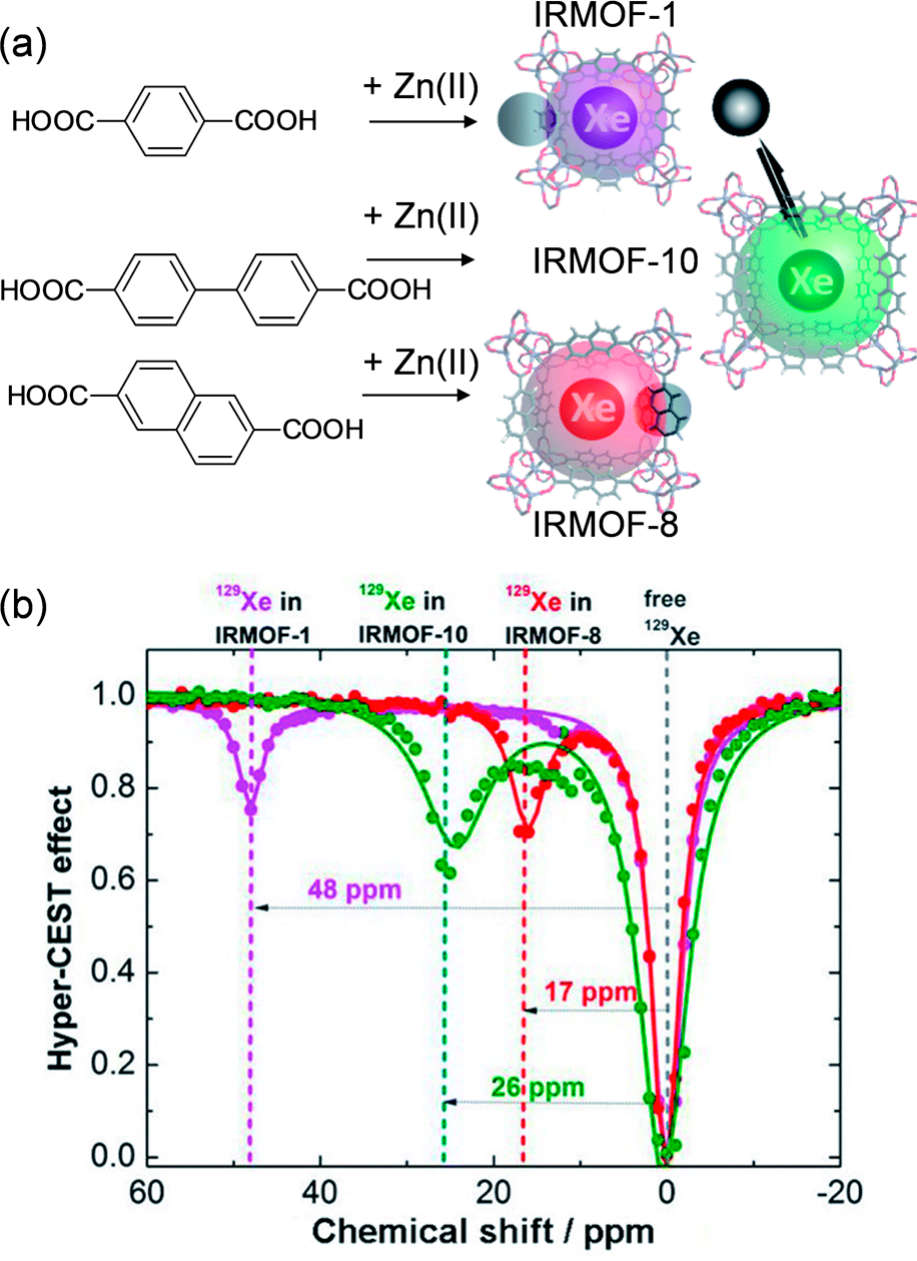
(a) Reaction scheme of the synthesis of IRMOF-1, IRMOF-10, and IRMOF-8 and (b) their frequency-dependent saturation spectra. To eliminate the influence of solvent, chemical shifts were referenced to the dissolved free ^129^Xe atom. Adapted with permission from ref (126). Copyright 2021, Royal Society of Chemistry.

Due to the sensitivity enhancement, hyperpolarized ^129^Xe MRI can be used for diagnosis of the respiratory system diseases.^124^ However, a detection of specific compounds in blood remains challenging due to the weak ^129^Xe signal in an aqueous solution. Here nanoparticles could play an important role as carriers of xenon. From this point of view, highly porous materials like MOFs seem to be perfect candidates to fulfill the task. If xenon is loaded inside MOF pores, its chemical shift is clearly distinguishable from that of free ^129^Xe in water, due to the surface and pore environment of the MOF, and thus such xenon nanocarriers can be detected. For instance, Zhou et al. studied the chemical shift of xenon when entrapped in pores of ZIF-8.^125^ Due to the hydrophobic pore environment, which offers specific interaction with the xenon atom, a significant chemical shift near 84 ppm was detected, which is ∼109 ppm apart from that of free ^129^Xe in aqueous solution (near 193 ppm). Moreover, the signal intensity of hyperpolarized ^129^Xe entrapped in MOF pores, corresponding to integral, was four times stronger than that of free ^129^Xe. In a follow-up work the authors studied the influence of a structure of MOF pores on MR properties of hyperpolarized ^129^Xe trapped inside these pores.^126^ A class of MOFs formed by similar octahedral Zn–O–C clusters and benzenecarboxylate ligands, namely IRMOF-1, IRMOF-8, and IRMOF-10 with pore diameters 7.93, 9.17, and 12.15 Ă, were selected. As expected by the authors, the ^129^Xe atom in each MOF produced an MR signal at its unique chemical shift - IRMOF-1 at 48 ppm, IRMOF-8 at 17 ppm, and IRMOF-10 at 26 ppm (Figure 13), and these irradiation differences were large enough to excite the signal from only one MOF under its particular frequency. The corresponding ultrasensitive MRI also showed a concentration-dependent intensity. Hence, the exploited MOF nanoparticles could be used as ultrasensitive MRI stains with diverse colors, making it possible to analyze complex samples qualitatively and quantitatively in the future.

### 4.2 MOFs and ^19^F MRI

One of the major drawbacks of traditional ^1^H MRI is the high natural background of ^1^H atoms impeding the accurate visualization of the contrast agent distribution. To address this problem, utilizing MRI based on a detection of fluorine has been proposed.^127^^19^F MRI “hot-spot” visualization of fluorinated tracers is an auspicious specific diagnostic method enabling very high contrast in MRI images due to the nearly zero fluorine background in the body. Furthermore, the resonance frequency of ^19^F is very close to that of ^1^H, allowing visualization of fluorinated tracers by commercial MRI scanners with only minor adjustments of the hardware and software. To employ ^19^F MRI in practice, fluorine probes are needed. These must contain a high content of fluorine atoms, which are chemically equivalent, have suitable relaxation times, adequate solubility in water, and an easily modifiable structure for targeting and biocompatibility/degradability.^127^ To prepare such agents, recently also MOF-based materials have been investigated.^128−130^

Wang et al. reported on a pH-responsive fluorinated ZIF for *in vitro* and *in vivo*^19^F MRI.^128^ ZIF-8 comprises Zn(II) ions and 2-methylimidazolate ligands and is known to be pH-responsive (stable at neutral and slightly basic conditions, but rather unstable in acidic conditions^131,132^). In this work, some of the ligands were exchanged to 4-(trifluoromethyl)imidazole to provide the fluorine moieties for the detection by ^19^F MRI. As expected, when the ^19^F nuclei were part of the framework, the peak intensity of fluorine signal ppm was very weak. However, the ^19^F MRI signal intensity could be turned-on by lowering the pH value to 5.5, which was attributed to the known disassembly of the nanoparticles at acidic conditions, and thus the ligand release. The half peak width of the fluorine probe was narrow indicating suitable T_2_ relaxation time which allows various MRI applications. Similarly, Yang et al. reported on a pH- and GSH-responsive ^1^H/^19^F bimodal MRI contrast agent, which was constructed by incorporating MnO_*x*_ into Zr-based MOF nanoparticles comprising tetrafluoroterephthalate ligands.^130^ Under an acidic environment, the nanoparticles disassembled releasing MnO_*x*_ and free fluorinated ligands. Due to the released of the ligands, ^19^F MRI signal was enhanced. Meanwhile, GSH, which is overexpressed in a tumor microenvironment, reduced MnO_*x*_ to Mn(II) ions, and thus, the T_1_-weighted MR imaging capability was improved.

## 5 Conclusions and Outlook

Considering the growing number of publications on MOFs for applications in MRI reported every year, there is no doubt that MOFs are extremely promising materials for developing novel contrast agents for MR imaging. Due to the versatile design possibilities of MOFs, materials with desired, precisely defined properties can be prepared. This offers opportunities for preparing not only agents for MRI, but also multifunctional responsive agents enabling multimodal imaging or combining imaging and drug delivery, i.e., theranostic agents, which are generally very challenging to synthesize but in great demand.

Although we have already learnt a lot about MOFs for applications in MRI over the past few years, there are still some issues that should be considered and addressed in order to facilitate the translation of such agents from “bench to bedside”. One of the most crucial points is bringing a clarity and standards to the material characterization. In order to evaluate and compare different materials, reported analytical data should be complete, including all measurement details. In the case of MR measurements, the following parameters must be given: solvent (water, buffer, gel, etc.), concentration, temperature, and also the field strength, relaxation times, and other relevant parameters (e.g., bandwidth, repetition time, echo time, temporal/spatial resolution, etc.). If relaxivities are determined, it should be clearly stated, whether they were calculated with respect to the molar concentration of the nanoparticles or of the metal ions. Moreover, it has been shown many times that the particle size and morphology also influenced the material MR properties significantly; therefore, the materials properties must be properly analyzed, and the corresponding analytical data must be provided as well as studies reporting on the material stability (in biological conditions) and toxicity (including the MOF building components). Last but not least, MRI *in vitro* studies should be carried out in simulated conditions, which are as close as possible to *in vivo* conditions.

Developing MRI contrast agents is a big endeavor. To maximize its efficiency, resources and success rate, a close collaboration between material scientists and clinicians is essential as well as employing innovative approaches and theoretical modeling, side by side with systematic investigations. Considering the MOFs unique features, which are not achievable with other materials, we have no doubts that the future of the research of MOFs for applications in MRI is bright and that great results can be expected.
